# Supplementary material for: Evaluation of a novel intervention providing insight into the tobacco industry to prevent the uptake of smoking in school-aged children: a mixed-methods study
Source: BMJ Open. 2017 Nov 3;7(11):e018031. doi: 10.1136/bmjopen-2017-018031 (PMC5695312; doi:10.1136/bmjopen-2017-018031)
Supplement: Supplementary file 1 [file bmjopen-2017-018031supp001.pdf]

Supplementary file 1: Outline of the Operation Smoke Storm intervention

**a) Year 7 component**

*Operation Smoke Storm* is a web-based educational package designed for delivery by teachers as part of a school's Personal, Social, Health and Economic Education (PSHE) curriculum. Teachers are provided with detailed lesson plans for three 50-minute classroom sessions (although the material can also be delivered as one longer session). Multimedia presentations, streamed 'live' over the Internet from *Kick It's* servers, are used to guide teachers and students through the lessons. Students act as secret agents to uncover the tactics of the tobacco industry and share what they find with others. The sessions also cover the health effects of tobacco, passive smoking, nicotine addiction and the economic cost of smoking. Sessions one and two include video clips followed by individual and group-based quizzes, and discussion activities where students learn about the harmful and addictive nature of smoking and methods used by tobacco companies to encourage young people to smoke. Students are provided with a workbook to record their answers. In session three, they then use this information to 'spread the word' in a group presentation to their class, in a medium of their choice such as through drama or song.

**b) Family component**

A ten-page A5 booklet accompanying the Year 7 lessons, designed to stimulate discussions about smoking between parents and students at home. The booklet contained the following interactive activities: 1) a repeat of the quiz questions students completed themselves in class to enable them to test their parents' knowledge about areas such as the health effects of smoking; 2) consideration of new information about the marketing practices employed by the tobacco industry, with particular focus on how

| |
|---|
| young people might be targeted; 3) an opportunity for students to give advice to other young people in various scenarios they might find themselves in relating to smoking. |
| **c) Year 8 booster component**<br>A one hour interactive session for 'off the shelf' delivery in Year 8 focusing on tobacco marketing strategies from the perspective of a tobacco industry executive and marketing company, as well as a health campaigner, both seen through the eyes of a teenager and reported direct to camera in the form of a social media blog. Students are asked a series of questions relating to the tobacco industry at key moments in the storyline. Two further optional activities are: 1) writing a slogan for a billboard poster, advertising a fake cigarette brand, in order to raise their awareness of how tobacco companies may portray smoking to young people; 2) writing a 'tweet' about the tobacco industry enabling students to reflect on their learning and consider their personal thoughts and feelings about this. |
