# Supplementary material for: Evaluation of a novel intervention providing insight into the tobacco industry to prevent the uptake of smoking in school-aged children: a mixed-methods study
Source: BMJ Open. 2017 Nov 3;7(11):e018031. doi: 10.1136/bmjopen-2017-018031 (PMC5695312; doi:10.1136/bmjopen-2017-018031)
Supplement: Supplementary file 2 [file bmjopen-2017-018031supp002.pdf]

Supplementary file 2: Themes and sub-themes identified in qualitative data from Year 7 and Year 8 students and teachers delivering to both groups

**Theme**	**Sub-themes**
Teachers' preparedness to deliver *Operation Smoke Storm**	• Previous experience teaching tobacco control<br>• Preparation before delivering the intervention<br>• Confidence to deliver the intervention<br>• Use of the teaching resources provided
Raised awareness	• New information learnt<br>- Prior knowledge; What's in a cigarette; Health effects; Tobacco industry; Understanding the message; Adding to knowledge learnt in Year 7 (booster)<br>• Impact of new information<br>- Realising the seriousness of smoking; Perceptions of smoking; Decision to Smoke; Using new information; Concerns and worries about others' smoking
Engagement with *Operation Smoke Storm*	• Views on the classroom-based sessions<br>- Messaging and storyline; Structure and timings; Formatting; Nature and variety of activities; Student ability<br>- Views on the booster session: Storyline and angle; Formatting; Following on from Year 7; Student ability; Nature and variety of activities<br>• Views on the family booklet<br>- How it was used; Discussions; Students' raised awareness of tobacco-related issues; Parental attitudes and raised awareness of tobacco-related issues; Timing
Extending *Operation Smoke Storm*	• E-cigarettes<br>- Students' knowledge; Discussions had in class; Students' perceptions and usage of e-cigs<br>• Peer pressure

*Data for this theme solely from teachers. Remaining themes represent data from students in Years 7 and 8 as well as teachers.
