# Supplementary material for: Evaluation of a novel intervention providing insight into the tobacco industry to prevent the uptake of smoking in school-aged children: a mixed-methods study
Source: BMJ Open. 2017 Nov 3;7(11):e018031. doi: 10.1136/bmjopen-2017-018031 (PMC5695312; doi:10.1136/bmjopen-2017-018031)
Supplement: Author's manuscript [file bmjopen-2017-018031.draft_revisions.pdf]

BMJ Open

BMJ Open is committed to open peer review. As part of this commitment we make the peer review history of every article we publish publicly available.

When an article is published we post the peer reviewers' comments and the authors' responses online. We also post the versions of the paper that were used during peer review. These are the versions that the peer review comments apply to.

The versions of the paper that follow are the versions that were submitted during the peer review process. They are not the versions of record or the final published versions. They should not be cited or distributed as the published version of this manuscript.

BMJ Open is an open access journal and the full, final, typeset and author-corrected version of record of the manuscript is available on our site with no access controls, subscription charges or pay-per-view fees (http://bmjopen.bmj.com).

If you have any questions on BMJ Open's open peer review process please email info.bmjopen@bmj.com

BMJ Open

Evaluation of a novel intervention providing insight into the tobacco industry to prevent the uptake of smoking in school-aged children: a mixed-methods study

For peer review only

Journal:	*BMJ Open*
Manuscript ID	bmjopen-2017-018031
Article Type:	Research
Date Submitted by the Author:	05-Jun-2017

Complete List of Authors:	Szatkowski, Lisa; University of Nottingham<br>Taylor, John; University of Nottingham<br>Fuller, Amy; University of Nottingham<br>Lewis, Sarah; University of Nottingham<br>Wu, Qi; University of York, Department of Health Sciences<br>Parrott, Steve; University of York<br>McNeill, Ann; King's College London, UK Centre for Tobacco Control Studies, National Addiction Centre, Institute of Psychiatry<br>Britton, John; University of Nottingham/ UK Centre for Tobacco and Alcohol Studies, Division of Epidemiology<br>Bauld, Linda; University of Stirling, Institute for Social Marketing<br>Jones, Laura; University of Birmingham, Public Health, Epidemiology & Biostatistics<br>Bains, Manpreet; University of Nottingham, Epidemiology and Public Health

**Primary Subject Heading**:	Addiction
Secondary Subject Heading:	Smoking and tobacco
Keywords:	PUBLIC HEALTH, Smoking, Prevention, Young people

SCHOLARONE™ Manuscripts

Evaluation of a novel intervention providing insight into the tobacco industry to prevent the uptake of smoking in school-aged children: a mixed-methods study

Lisa Szatkowski[1], John Taylor[1], Amy Fuller[1], Sarah Lewis[1], Qi Wu[2], Steve Parrott[2], Ann McNeill[3], John Britton[1], Linda Bauld[4], Laura L Jones[5], Manpreet Bains[1]

[1,2,3,4,5]UK Centre for Tobacco and Alcohol Studies;

[1]University of Nottingham, Division of Epidemiology and Public Health, Clinical Sciences Building, Nottingham City Hospital, Nottingham, UK, NG5 1PB;

[2]Department of Health Sciences, University of York, Seebohm Rowntree Building, Heslington, York, UK, YO10 5DD;

[3]King's College London, National Addictions Centre, Institute of Psychiatry, Psychology and Neuroscience, Addiction Sciences Building, 4 Windsor Walk, Denmark Hill, London, UK, SE5 8BB;

[4]Institute for Social Marketing, 3Y1, University of Stirling, Stirling, UK, FK9 4LA;

[5]Public Health, Epidemiology and Biostatistics, School of Health and Population Sciences, College of Medical and Dental Sciences, University of Birmingham, Edgbaston, Birmingham, UK, B15 2TT.

Correspondence to: Dr Manpreet Bains: manpreet.bains@nottingham.ac.uk

For peer review only

For peer review only - http://bmjopen.bmj.com/site/about/guidelines.xhtml

ABSTRACT

Objectives: Evidence from the US Truth® campaign suggests that interventions focusing on tobacco industry practices and ethics may be effective in preventing youth smoking uptake. We developed, piloted and evaluated a school-based intervention based on this premise.

Methods: Exploratory study Students in Years 7–8 (aged 11–13) in two UK schools received *Operation Smoke Storm*, comprising three 50-minute classroom-based sessions in Year 7, an accompanying family booklet and a 1-hour classroom-based booster session in Year 8. We compared the risk and odds of ever smoking and susceptibility to smoking in Year 8 students in study schools post-intervention compared with students in control schools. Focus groups and interviews with students, teachers and parents evaluated the acceptability of the intervention.

Results: In intervention schools the combined prevalence of ever smoking and susceptibility increased from 18.2% in Year 7 to 33.8% in Year 8. There was no significant difference in the odds of a Year 8 student in an intervention school being an ever smoker or susceptible never smoker compared with controls [adjusted OR 1.28, 95%CI 0.83-1.97, p=0.263] and no significant difference in the odds of ever smoking (aOR 0.82, 95%CI 0.42-1.58, p=0.549). Teachers highlighted differences by academic ability in how well the messages presented were understood. Use of the family component was low but was received positively by parents who engaged with it.

For peer review only - http://bmjopen.bmj.com/site/about/guidelines.xhtml

Conclusions: *Operation Smoke Storm* is an acceptable resource for delivering smoking-prevention education but it does not appear to have reduced smoking and susceptibility.

For peer review only

For peer review only - http://bmjopen.bmj.com/site/about/guidelines.xhtml

Strengths and limitations

- There is little conclusive evidence that school-based smoking prevention interventions can have long-term impacts on students' smoking behaviours.
- Evidence from the United States suggests that mass-media smoking prevention campaigns focusing on tobacco industry practices and ethics may be effective in preventing youth smoking uptake, but this approach has not been tested in a school setting.
- This study suggests that delivery of such a school-based smoking prevention intervention in the early years of secondary education (aged 11-13) is feasible, well-received by students, teachers and parents, and may prompt some positive changes in knowledge and attitudes towards smoking.
- However, the intervention did not have a significant impact on self-reported ever smoking and susceptibility to smoking.

For peer review only

For peer review only - http://bmjopen.bmj.com/site/about/guidelines.xhtml

INTRODUCTION

In the UK, nearly 40% of adult smokers start to smoke regularly before the age of 16[1] and those who start at an early age are more likely to die from a smoking-attributable cause[2]. Therefore, preventing young people from smoking is an important public health priority and school-based approaches provide an opportunity to reach large numbers of young people. However, there is little conclusive evidence that school-based prevention interventions have anything beyond short-term effects[3–5].

In the United States the mass media *Truth*® campaign has demonstrated some success in encouraging young people not to smoke, focusing on the ethics and exploitative tactics of the tobacco industry[6–8]. Its acceptability and effectiveness has been recognised as worth exploring further in school settings[5]. Previously we have reported results of a preliminary qualitative evaluation amongst Year 7 students (aged 11-12) in two UK schools of the acceptability of a novel school-based intervention, *Operation Smoke Storm* (OSS), based on the premise of *Truth*®[9]. Initially, *OSS* comprised three 50-minute multimedia interactive teaching sessions, developed by *Kick It*, who deliver the National Health Service (NHS) Stop Smoking Service for several London boroughs[10]. Further description of this intervention is given in supplementary file 1.

In focus groups conducted after the delivery of *OSS* students reported enjoying the intervention and acquiring new knowledge about smoking and the tobacco industry, which seemed to strengthen their aversion to smoking[9]. In one-to-one interviews teachers expressed confidence delivering the ‘off the shelf’ resource, although they

For peer review only - http://bmjopen.bmj.com/site/about/guidelines.xhtml

highlighted a need for the package to be flexible and not dependent on lesson length, teacher confidence or expertise[9]. Following this feedback, Year 7 lessons were refined by the research team alongside *Kick It*, primarily to correct technical issues and to increase flexibility and provide teachers with more guidance to help them facilitate discussions regardless of their own level of knowledge. The intervention was also extended to include a family booklet to complement the Year 7 lessons to encourage parents to talk to their children about smoking and a 'booster' session for use with Year 8 students (aged 12-13) to reinforce the anti-smoking message. These family and booster components are described in supplementary file 1. Here we report quantitative and qualitative data evaluating the acceptability and effectiveness of the full intervention package.

METHODS

Quantitative evaluation

Collection of baseline and follow-up data

The recruitment process and characteristics of the two schools where *OSS* was delivered are described elsewhere[9]. PSHE teachers delivered the first intervention component to all Year 7 students in both schools (n=585) in autumn 2013. Before and after intervention delivery all students were asked to complete an anonymous questionnaire to gather information on their socio-demographic characteristics as well as smoking behaviours and attitudes. Students were asked if they had ever smoked, as

For peer review only - http://bmjopen.bmj.com/site/about/guidelines.xhtml

well as a set of three previously-validated questions to assess their susceptibility to smoking[11]. Students were classified as non-susceptible if they answered 'no' to the question 'do you think that you will try a cigarette soon?' and 'definitely not' to the questions 'if one of your best friends were to offer you a cigarette, would you smoke it?' and 'do you think you will smoke a cigarette at any time during the next year?' Students who answered 'definitely yes', 'probably yes' or 'probably not' to either of the last two questions or 'yes' to the first question were classified as susceptible.

One year later, in autumn 2014, the booster session was delivered to the same students, then in Year 8 (n=538). In one school PSHE specialists delivered the booster; 40-minute lessons meant they needed two sessions to cover the material. In the second school, changes in the organisation of PSHE meant that the booster was instead delivered by science teachers; lessons here were one hour in length and the material was delivered in a single session. An anonymous questionnaire was administered after the booster session to gather data on smoking behaviours and attitudes and socio-demographic characteristics.

In autumn 2014 the refined Year 7 intervention component was also delivered to the new cohort of Year 7 students (n=350) in one school only, and these students were given the new family booklet to take home. Changes in the delivery of PSHE in the second school meant that they were not able to accommodate delivery of the Year 7 sessions. Questionnaire data were collected at the end of the sessions to gain information about the acceptability of the revised intervention and family component.

Collection of control data from a non-randomised comparison group

For peer review only - http://bmjopen.bmj.com/site/about/guidelines.xhtml

Given some difficulty in recruiting schools, and in order to minimise costs, we chose to use external control data collected as part of another study just prior to ours. The Nottingham School Smoking Survey collected data from students in eight schools local to the study area in Spring 2011, 2012 and 2013 (though not all schools participated in every wave). The primary aim of this survey was to evaluate changes in young people's smoking behaviour following the introduction of point-of-sale tobacco display legislation[12,13]. By mid-2013 data were available on current smoking and susceptibility to smoking in Year 7 and Year 8 for two successive cohorts of students (i.e. students who were in Year 7 in 2011 and Year 8 in 2012 and students who were in Year 7 in 2012 and Year 8 in 2013).

Statistical analysis

All data management and analysis was carried out using Stata v13 (StataCorp, College Station, TX). Logistic regression was used to compare the self-reported odds of a combined outcome of ever smoking and susceptibility to smoking, plus ever smoking on its own, in Year 8 students after the delivery of the booster session to the odds amongst Year 8 students in the two combined cohorts of students in control schools, using a multilevel model to adjust for clustering with the effect of school modelled as a random intercept. Due to difficulties in linking students' responses to the Year 7 and Year 8 questionnaires in intervention schools, odds ratios could not be adjusted for differences between intervention and control groups at baseline. However, models were adjusted for socio-demographic variables using data collected in Year 8 and smoking behaviour at Year 7 was compared between intervention and

For peer review only - http://bmjopen.bmj.com/site/about/guidelines.xhtml

control schools to quantify any differences. Unfortunately, a comparable measure of deprivation was not available across intervention and control schools. Therefore, a proxy indicator of deprivation was created, considering students in the most deprived quintile of the Index of Multiple Deprivation (IMD) in the control schools and those who reported being eligible for free school meals in the intervention schools as deprived relative to all others. Given the exploratory nature of the study, we have not applied a correction for multiple hypothesis testing but, instead, have presented results with 95% confidence intervals (95% CIs) and p-values in order to allow the reader to evaluate the findings fully. We also calculated unadjusted and adjusted risk differences (using the 'adjrr' post-estimation command in Stata) to compare intervention and control schools.

The non-randomised study was not intended to be fully powered but was instead planned as an exploratory study of the potential effectiveness of the intervention. A pre-study power calculation, based on estimates of the likely achieved sample size in intervention and control schools and the self-reported prevalence of ever smoking and susceptibility amongst Year 8 students, suggested that we would be able to estimate the risk difference to within 6.6% i.e. if the observed effect was 6.7% or greater the confidence intervals would preclude the possibility of no effect or a negative effect of the intervention. This effect size was consistent with the size of effect that a subsequent cluster-randomised controlled trial would be powered to detect, and in line with the size of effect used to power the ASSIST study[14]. For each of our outcomes (ever smoking and susceptibility to smoking, plus ever smoking on its own) we also calculated Bayes factors under three different scenarios in order to assess whether our data provided substantial evidence for or against the null hypothesis: 1) assuming a

For peer review only - http://bmjopen.bmj.com/site/about/guidelines.xhtml

maximum odds ratio of 2 i.e. a doubling of never smokers in intervention compared to control schools, taking hypothesised values uniformly distributed between 0 and the maximum as plausible values; 2) assuming a plausible predicted odds ratio of 2 and taking hypothesised values in a normal distribution around this value; 3) assuming a plausible predicted odds ratio of 2 and taking hypothesised values in a half normal distribution around this value. A Bayes factor of 3 or more was taken as substantial evidence against the null hypothesis and 1/3 or less as evidence for the null.

We have followed the STROBE statement in reporting the results of this study.

Qualitative evaluation

Focus group and interview procedures

The qualitative evaluation comprised focus groups with Year 7 and Year 8 students, interviews with teachers who delivered the Year 7 sessions and the Year 8 booster session, and paired Year 7 student-parent interviews to evaluate the family booklet, each guided by a semi-structured interview schedule. We used the same procedures as described previously[9]. In summary, we conducted two gender-specific focus groups with Year 7 students in the one school that delivered the revised sessions (16 students in total – 8 male, 8 female) and eight focus groups with Year 8 students across the two schools (51 students in total – 25 male, 26 female). Students shared their views on the sessions and their awareness of and attitudes towards the tobacco industry and smoking. Both Year 7 focus groups lasted for 26 minutes and Year 8 focus groups

For peer review only - http://bmjopen.bmj.com/site/about/guidelines.xhtml

lasted for 24 minutes on average (range 11–35 minutes). All Year 7 and Year 8 teachers who delivered part of the intervention were invited by email to be interviewed about its acceptability and effectiveness; ten Year 7 teachers and six Year 8 teachers took part (four from School 1, two from School 2, interviews lasted 26 minutes on average [range 19-33 minutes]). The family booklet was accompanied by a letter inviting parents to express an interest in participating in a paired student-parent interview to explore their views. These interviews sought students' and parents' views on the booklet and how they engaged with it. An inconvenience allowance (£15 high-street voucher) was offered to each pair who participated (n = 9). Interviews took place in participants' home or on school premises according to individual preference (lasted 23 minutes on average and ranged between 13 – 33 minutes).

Data analysis

Analysis procedures were similar to those used previously[9], which followed the framework approach[15,16]. Digital audio-recordings were transcribed verbatim. A sample of focus group and interview transcripts was read initially (by AT and JT) to identify initial codes, themes and sub-themes and any within- or between-group differences (school and gender). As in our earlier work, codes identified from the focus groups, teacher interviews and student-parent interviews were similar (apart from teachers' interview data identifying a theme about preparation to deliver the intervention) and thus all Year 7 data were analysed together and similarly all Year 8 data. Initial themes and sub-themes were discussed between the researchers (AT, JT, MB, LS) to reach consensus on an initial analytical framework. This framework was

For peer review only - http://bmjopen.bmj.com/site/about/guidelines.xhtml

applied and refined following analysis of the remaining transcripts and until the point of data saturation. Data were then indexed according to the final framework and charted into matrices according to each theme to facilitate synthesis and interpretation.

Similar themes were identified for both the Year 7 and Year 8 intervention components, and these supported those reported in our initial evaluation[9]: *Teachers' preparedness to deliver OSS*; *Raised awareness*; *Engagement with the intervention*; and *Options for extending the resource* (see supplementary file 2 for details of themes). Qualitative findings with respect to the Year 7 sessions were similar to those reported previously[9] and the amendments made to correct technical issues, increase flexibility and provide teachers with more guidance were positively received. Therefore, the qualitative findings presented here focus on evaluation of the family booklet and Year 8 booster session.

Ethics and consent

The study received ethical approval from the University of Nottingham Medical School Research Ethics Committee (reference 13122012 CHS EPH Smoking). Parents of students in both Year 7 and Year 8 were sent a letter informing them about *OSS* and the accompanying academic evaluation, approximately three weeks prior to delivery. They were asked to return an opt-out slip if they did not want their child to complete a questionnaire or to participate in a focus group. Students were able to opt out of questionnaire completion and were under no obligation to volunteer for focus groups. Written informed consent was obtained from participants prior to data collection.

For peer review only - http://bmjopen.bmj.com/site/about/guidelines.xhtml

RESULTS

Did *OSS* have an impact on smoking behaviour?

Completed questionnaires were received from 445 Year 8 students in intervention schools and 1,692 Year 8 students in control schools; Table 1 describes students' characteristics.

As expected, given the non-randomised nature of the study there were significant differences between students in intervention and control schools. In control schools a greater proportion of students were of non-white ethnicity, had parents who smoked, reported smoking was allowed in their home, had more friends who smoked and were ever smokers themselves.

Table 2 shows the odds of a student being a susceptible never smoker and/or an ever smoker in Year 7 and Year 8 in the two intervention schools compared to control schools. After adjusting for significant confounders, there were no differences in ever smoking and susceptibility to smoking between intervention and control schools in Year 7. In Year 8, after adjusting for significant confounders, the odds of a student in an intervention school being an ever smoker or susceptible never smoker were 28% higher than the odds for a student in a control school, though this difference was not statistically significant (adjusted OR 1.28, 95% CI 0.83-1.97, p=0.263). The adjusted risk difference suggested a non-significant 4.1% higher prevalence of ever smoking and susceptibility to smoking in intervention schools. Students in intervention schools were slightly less likely to have ever smoked compared to students in control schools, though again the difference was not statistically significant (adjusted OR 0.82, 95%

For peer review only - http://bmjopen.bmj.com/site/about/guidelines.xhtml

CI 0.42-1.58, p=0.549). The adjusted risk difference suggested a non-significant 2.0% lower prevalence of ever smoking in intervention schools.

Bayes factors for the combined outcome were 1/3 or lower under each of the three scenarios tested, suggesting that our data provide substantial evidence for the null hypothesis of no positive effect of the intervention. Bayes factors for ever smoking were all close to one, suggesting that our data are insensitive and unable to distinguish between the alternative and null hypotheses.

What did students, teachers and parents think about *OSS*?

Students broadly liked *OSS*; 77.1% of Year 7 students said that the revised Year 7 sessions were very good or okay and 72.4% of Year 8 students evaluated the booster session similarly. Qualitative data from Year 8 focus groups showed the booster session was well received and that most students bought into the storyline (Table 3a,b).

Of the 61.6% of Year 7 students who reported receiving the family booklet and taking it home, 43.0% said they showed it to their mother or another adult female, 21.5% reported showing to their father or another adult male and 24.4% said that they did not show the booklet to anyone. Very few reported having completed activities with a parent or carer. Even though Year 7 students and parents who were interviewed endorsed the family booklet as a way to improve knowledge and initiate conversations around smoking (Table 3c,d), our qualitative data also indicated that often the booklet was not used as intended – many students simply did not show the booklet to their parents or realise the booklet was for them to complete with their parents (Table 3e,f).

For peer review only - http://bmjopen.bmj.com/site/about/guidelines.xhtml

Did *OSS* change students' knowledge and attitudes about smoking?

69.3% of Year 7 students and 45.0% of Year 8 students thought that *OSS* had made it less likely that they would ever try a cigarette. Students displayed some changes in knowledge and attitudes over the course of the study (Table 4).

Qualitative findings from Year 8 students and teachers suggest the booster session raised awareness of the harmful effects of tobacco and some students showed an appreciation of why and how the tobacco industry might target young people (Table 5a,b). However, teachers mentioned that not all students understood this message and highlighted differences in the extent to which students of higher and lower academic abilities could remember the new information and complete the activities (Table 5c,d).

DISCUSSION

This project was the first to formally evaluate a school-based smoking prevention intervention highlighting the ethics and exploitative tactics of the tobacco industry. The intervention was feasible to deliver in the classroom, was generally acceptable to teachers, students and parents and helped to raise awareness about smoking-related issues and the tobacco industry. However, there was no significant difference in the odds or risk of self-reported ever smoking and susceptibility to smoking in students who received *OSS* compared to students from local schools where the intervention was not delivered.

For peer review only - http://bmjopen.bmj.com/site/about/guidelines.xhtml

Synthesis of the quantitative and qualitative data offers potential suggestions as to why the intervention does not appear to be effective in preventing smoking uptake. In both the focus groups with Year 7 students reported previously[9], and those following delivery of the revised Year 7 sessions, students' interest and recall centred mainly on the chemical constituents of cigarettes and/or the health effects of smoking. There was some suggestion from teachers that concepts relating to tobacco marketing, particularly where they were mentioned more subtly, were too advanced for students of lower academic ability to fully grasp. Given that educational attainment is inversely associated with adolescent smoking[18], it might be that *OSS* failed to reach those students most likely to become smokers.

The prevalence of smoking amongst young people increases with age[19] and it might be that any effect of *OSS* on uptake is delayed beyond the follow-up period studied here. Many students reported that participation in *OSS* had made it less likely that they would try a cigarette, and there was evidence of increasing disagreement over time with statements such as 'smoking is not that serious compared with other drugs that young people use'. These data are encouraging and, although these shifts in attitudes are not reflected in self-reported smoking and susceptibility in Year 8, the possibility remains that the impact of the intervention may become evident among these students in years to come.

The Year 8 students on whom the primary analysis is based received the original version of the Year 7 lessons that were subsequently revised. Therefore it is possible that the effect of the revised resources on smoking and/or susceptibility might have

For peer review only - http://bmjopen.bmj.com/site/about/guidelines.xhtml

been different. However, given the fact that the majority of the changes made were to correct technical issues rather than changes to content, this is unlikely. In addition, Year 8 students had not received the family component of the intervention. However, few Year 7 students in the second phase of the study used the booklet as intended so it is unlikely that this would have a substantial effect on the outcomes.

The 95% CIs around the odds ratios quantifying differences in smoking behaviours between students in intervention and control schools were wide, and the adjusted risk differences were small. The direction of the point estimate for the odds of ever smoking tentatively suggests that exposure to *OSS* might reduce the odds of this outcome, although the odds ratio for the combined outcome of ever smoking plus susceptibility suggests an increase in odds. A reduction in ever smoking following exposure to *OSS* would be encouraging, and with a larger sample size the precision of the effect estimates would improve and smaller effect sizes may be detected as statistically significant.

The study findings are based on data from only two schools and may not be generalisable to schools more widely, particularly with regard to students' ethnicity and deprivation. The non-randomised comparison meant there were significant differences between the characteristics of students in intervention and control schools, which we were not able to adjust for. Our conclusions also rely on self-reported data, even though measures such as ensuring students' anonymity were in place to encourage honest responses.

For peer review only

The use of topic guides and the rigorous analytical process of the framework approach counterbalanced any potential for biased interpretation in favour of the intervention. However, some Year 8 focus groups had a small number of participants meant there was a less than ideal group dynamic. Finally, the students, teachers and parents who took part in the focus groups and interviews were a self-selecting sample, which introduced potential for bias.

Despite there being no evidence of effectiveness in this study, there is scope for further work to understand whether the concept behind *OSS* is worth pursuing further. *OSS* as it stands is probably not suitable for use with students older than the Year 7 and Year 8 groups, but the concept might be effective if used as the basis of an age-appropriate intervention with older students who might be better able to engage with subtle messages about industry influences. Alternatively, *OSS* might usefully be adapted to include fully differentiated activities and resources for use with different academic abilities. Given the erosion of PSHE within the curriculum, there is scope to understand whether *OSS* could be delivered effectively in other settings such as youth groups. Finally, further work is warranted to explore how to effectively engage parents and guardians more in supporting their child to remain smoke free.

DECLARATIONS

Acknowledgements: We thank the PSHE teaching leads, teachers and students at the schools who took part in this study, as well as the chair and members of the Study Steering Committee and Data Monitoring and Evaluation Committee for their

For peer review only - http://bmjopen.bmj.com/site/about/guidelines.xhtml

For peer review only

valuable advice and support. We also thank Tomasz Letniowski and Toby Fairs-Billam at Kick It who developed *OSS* but who were independent of this evaluation.

Authors' contributions: LS, MB, SL, AM, JB, LLJ, LB, SP and QW designed the study. JT and AT led the focus groups and conducted the teacher interviews; MB, LS and LLJ observed the focus groups. JT and AT analysed the data with MB providing external validation of themes. LS and JT wrote the first draft of this manuscript. All authors made critical comments on subsequent drafts of the paper and have approved the final version.

Data sharing: Requests for access to data should be addressed to the corresponding author.

Funding: This project was funded by the National Institute for Health Research Public Health Research (NIHR PHR) Programme [grant number 11/3010/02]. The views and opinions expressed herein are those of the authors and do not necessarily reflect those of the NIHR PHR Programme or the Department of Health. The authors are members of the UK Centre for Tobacco and Alcohol Studies (UKCTAS), a UK Clinical Research Collaboration (UKCRC)-funded Public Health Research Centre of Excellence. Funding from the British Heart Foundation, Cancer Research UK, the Economic and Social Research Council, the Medical Research Council and the National Institute of Health Research, under the auspices of the UK Clinical Research Collaboration, is gratefully acknowledged.

For peer review only - http://bmjopen.bmj.com/site/about/guidelines.xhtml

Competing interests: Laura Jones receives personal fees from the National Centre for Smoking Cessation and Training, outside the submitted work. There are no other competing interests to report.

REFERENCES

1. Dawe F. *General Lifestyle Survey Overview. A Report of the 2011 General Lifestyle Survey*. Newport: Office for National Statistics; 2013. http://www.ons.gov.uk/ons/dcp171776_302558.pdf.

2. Department of Health. *Smoking Kills: A White Paper on Tobacco*. London: Department of Health; 1998. http://www.archive.official-documents.co.uk/document/cm41/4177/4177.htm.

3. Thomas R, McLellan J, Perera R. School-based programmes for preventing smoking. *Cochrane Database Syst Rev*. 2013;4(CD001293). doi:10.1002/14651858.CD001293.pub3.

4. Wiehe S, Garrison M, Christakis D, Ebel B, Rivara F. A systematic review of schoolbased smoking prevention trials with long-term follow-up. *J Adolesc Health*. 2005;36:162-169. doi:10.1016/j.jadohealth.2004.12.003.

5. National Institute for Health and Clinical Excellence. *School-Based Interventions to Prevent the Uptake of Smoking among Children and Young People (NICE Public Health Guidance 23)*. London: National Institute for Health and Clinical Excellence; 2010. http://www.nice.org.uk/guidance/PH23.

6. The truth® campaign. http://www.protectthetruth.org/truthcampaign.htm. Accessed September 12, 2016.

7. Farrelly M, Healton C, Davis K, Messeri P, Hersey J, Haviland M. Getting to the Truth: Evaluating National Tobacco Countermarketing Campaigns. *Am J Public Health*. 2002;92:901-907.

8. Farrelly M, Davis K, Haviland M, Messeri P, Healton C. Evidence of a dose-response relationship between "truth" antismoking ads and youth smoking prevalence. *Am J Public Health Bull Edinb*. 2005;95:425-431. doi:10.2105/AJPH.2004.049692.

9. Taylor J, Taylor A, Lewis S, et al. A qualitative evaluation of a novel intervention using insight into tobacco industry tactics to prevent the uptake of smoking in school-aged children. *BMC Public Health*. 2016;16:539. doi:10.1186/s12889-016-3205-8.

For peer review only - http://bmjopen.bmj.com/site/about/guidelines.xhtml

10. Kick It. https://www.kick-it.org.uk/. Accessed September 12, 2016.

11. Pierce J, Choi W, Gilpin E, Farkas A, Merritt R. Validation of susceptibility as a predictor of which adolescents take up smoking in the United States. *Health Psychol*. 1996;15(5):355-361. doi:10.1037/0278-6133.15.5.355.

12. Spanopoulos D, Britton J, McNeill A, Ratschen E, Szatkowski L. Tobacco display and brand communication at the point of sale: implications for adolescent smoking behaviour. *Tob Control*. 2014;23(1):64-69. doi:10.1136/tobaccocontrol-2012-050765.

13. Bogdanovica I, Szatkowski L, McNeill A, Spanopoulos D, Britton J. Exposure to point-of-sale displays and changes in susceptibility to smoking: findings from a cohort study of school students. *Addict Abingdon Engl*. 2015;110(4):693-702. doi:10.1111/add.12826.

14. Campbell R, Starkey F, Holliday J, et al. An informal school-based peer-led intervention for smoking prevention in adolescence (ASSIST): a cluster randomised trial. *The Lancet*. 2008;371(9624):1595-1602. doi:10.1016/S0140-6736(08)60692-3.

15. Ritchie J, Lewis J, Elam G. *Qualitative Research Practice: A Guide for Social Science Students and Researchers.* London: Sage; 2003.

16. Gale NK, Heath G, Cameron E, Rashid S, Redwood S. Using the framework method for the analysis of qualitative data in multi-disciplinary health research. *BMC Med Res Methodol*. 2013;13:117. doi:10.1186/1471-2288-13-117.

17. Hanewinkel R, Isensee B, Sargent JD, Morgenstern M. Cigarette advertising and adolescent smoking. *Am J Prev Med*. 2010;38(4):359-366. doi:10.1016/j.amepre.2009.12.036.

18. Amos A, Angus K, Bostock Y, Fidler J, Hastings G. *A Review of Young People and Smoking in England*. Public Health Research Consortium; 2009. http://phrc.lshtm.ac.uk/papers/PHRC_A7-08_Final_Report.pdf.

19. Fuller E (ed). *Smoking, Drinking and Drug Use among Young People in England in 2014*. Leeds: Health and Social Care Information Centre; 2015. http://www.hscic.gov.uk/catalogue/PUB17879/smok-drin-drug-youn-peop-eng-2014-rep.pdf.

For peer review only

For peer review only - http://bmjopen.bmj.com/site/about/guidelines.xhtml

Table 1: Characteristics of Year 8 students in intervention and control schools

	Intervention schools, n (%)	Control schools, n (%)	p-value for difference[a]
**Total number of completed questionnaires received**	445	1692	
**Sex**			
Male	200 (44.9)	843 (49.8)	0.482
Female	216 (48.5)	843 (49.8)	
Missing	29 (6.5)	6 (0.4)	
**Ethnic group**			
White	368 (82.7)	1309 (77.4)	<0.001
Non-white	27 (6.1)	220 (13.0)	
Missing	50 (11.2)	163 (9.6)	
**Parental smoking**			
Neither	302 (67.9)	1123 (66.4)	0.031
At least one	106 (23.8)	516 (30.5)	
Missing	37 (8.3)	53 (3.1)	
**Sibling smoking**			
None	365 (82.0)	1461 (86.4)	0.852
At least one	43 (9.7)	178 (10.5)	
Missing	37 (8.3)	53 (3.1)	
**Smoking in the home**			
Not allowed	369 (82.9)	1460 (80.4)	<0.001
Allowed	36 (7.6)	375 (16.3)	
Missing	42 (9.4)	57 (3.4)	
**Number of friends who smoke**			
None	289 (64.9)	734 (43.4)	<0.001
One or two	48 (10.8)	236 (14.0)	
Three or more	18 (4.0)	254 (15.0)	
Missing	90 (20.2)	468 (27.7)	
**Rebelliousness and sensation seeking**[17]			
Low	225 (50.6)	870 (51.4)	0.661
High	176 (39.6)	715 (42.3)	
Missing	44 (9.9)	107 (6.3)	
**Academic performance (self-perceived)**			
Excellent or good	313 (70.3)	1228 (72.6)	0.372
Average or below average	92 (20.7)	406 (24.0)	
Missing	40 (9.0)	58 (3.4)	
**Eligible for free school meals**			
No	374 (84.0)	Not collected	N/A
Yes	25 (5.6)		
Missing	46 (10.3)		
**Index of Multiple Deprivation (IMD) quintile**			
Least deprived	Not collected	375 (22.2)	N/A
2		160 (9.5)	
3		282 (16.7)	
4		240 (14.2)	
Most deprived		261 (15.4)	
Missing		374 (22.1)	

[a] excluding missing data

Table 2: Odds ratios and adjusted risk differences for smoking outcomes in intervention compared to control schools

	Prevalence (%)		Unadjusted			Adjusted*		
	**Students in intervention schools**	**Students in control schools**	**Odds of outcome in intervention vs. control schools OR (95% CI)**	**p-value**	**Risk difference % (95% CI)**	**Odds of outcome in intervention vs. control schools OR (95% CI)**	**p-value**	**Risk difference % (95% CI)**
**Year 7 (before intervention delivery)**								
**Ever smoker or susceptible never smoker**	18.2 (92/505)	22.9 (351/1530)	0.82 (0.43-1.55)	0.536	-4.7 (-15.3-5.9)	1.74 (0.54-5.56)	0.351	5.9 (-13.8-2.6)
**Ever smoker**	2.4 (12/505)	6.4 (98/1530)	0.38 (0.13-1.08)	0.070	-4.0 (-6.9-1.2)	1.22 (0.13-11.3)	0.858	0.4 (-9.9-10.8)
**Year 8 (after intervention delivery)**								
**Ever smoker or susceptible never smoker**	33.8 (145/429)	30.9 (504/1631)	1.17 (0.70-1.95)	0.556	2.9 (-4.0-9.8)	1.28 (0.83-1.97)	0.263	4.1 (-0.5-8.6)
**Ever smoker**	7.9 (34/429)	10.7 (175/1631)	0.80 (0.32-1.98)	0.622	-2.8 (-7.8-2.1)	0.82 (0.42-1.58)	0.549	-2.0 (-5.4-1.4)

*Adjusted for: perceived academic ability; rebelliousness; sibling smoking; parental smoking; and whether smoking is allowed in the family home.

For peer review only

Table 3: What did students, teachers and parents think about *OSS*?

a) *It was really good. It was something different and I liked it.* (School 2, F)

b) *They're [the videos] really cool because I like when that girl went on a mission. That was, kind of like, interesting because I was like, "What is she going to do next?* (School 1, F)

c) *I learned something, I didn't know about all the additives if you like; and the sneaky way that the big companies and the amount of money involved and all of that really.* (School 1, Parent 1)

d) *We've discussed it since and had a chat about it. We were talking about it the other day, weren't we, things like the booklet and things like that and talking about what we now know about it. It was building on really what you'd done in [Drug Abuse Resistance Education] DARE at primary, wasn't it, just taking it a bit further*. (School 1, Parent 7)

e) *My tutor didn't really explain what it actually was about, so I didn't know I actually had to do anything with it, that's why I didn't show my mum.* (School 1, Parent 3)

f) *That's why I just thought, "oh, it's for my parents, it's not for me."* (School 1, F)

For peer review only

Table 4: Mean Likert scale responses (1=strongly agree, 5=strongly disagree)

	How far do you agree with the following statements? (mean+SD for statements 1-3; median+IQR for statement 4)			p-value*
	**Baseline**	**After Year 7 lessons in Phase 1**	**After Year 8 lessons in Phase 2**	
1) Companies that make cigarettes only try to attract customers aged 18+	2.30 (1.04)	2.85 (1.22)	3.47 (1.07)	<0.001
2) Companies that make cigarettes sell dangerous products, but still operate in a fair and decent way	2.79 (0.95)	2.80 (1.04)	2.95 (0.95)	0.030
3) Smoking is not that serious compared with other drugs young people use	3.06 (1.13)	3.20 (1.16)	3.24 (1.09)	0.034
4) Nicotine in cigarettes is one of the most addictive drugs that people use	2 (1-3)	2 (1-2)	2 (1-3)	<0.001

*ANOVA F test for normally distributed variables, Kruskal-Wallis test for non-normally distributed variables

For peer review only

Table 5: Did *OSS* change students' knowledge and attitudes about smoking?

a) *I didn't know about like all the effects until this year, and it's just like, it just shows you what actually smoking does. It just opened my eyes a bit.* (School 1, F)

b) *If they target to young people and try and get to young people, then they will get more money, 'cause there'll be more people getting addicted to it.* (School 2, F)

c) *Do you know that little clip where the boss is being very subtle going, 'oh do you use social media?'. And, 'oh we could do brand placement. Oh but we're not allowed to.' And it was all very subtle…Yeah and do you know lower-ability pupils wouldn't have got that. I think that would have confused them, where the other pupils it wouldn't have.* (School 1, Teacher 1)

d) *So whenever they talked about like their Tweets for social media, they kind of went for, 'You shouldn't smoke, it's bad for you. You shouldn't smoke. Cigarettes have got all this stuff in them,' so kind of the obvious stuff from it, but they then don't take it that step further to think, like, should they be publicising it, yeah, taking that conversation a bit deeper.* (School 1, Teacher 2)

For peer review only

Supplementary file 1: Outline of the Operation Smoke Storm intervention

a) Year 7 component
Operation Smoke Storm is a web-based educational package designed for delivery by teachers as part of a school's Personal, Social, Health and Economic Education (PSHE) curriculum. Teachers are provided with detailed lesson plans for three 50-minute classroom sessions (although the material can also be delivered as one longer session). Multimedia presentations, streamed 'live' over the Internet from *Kick It's* servers, are used to guide teachers and students through the lessons. Students act as secret agents to uncover the tactics of the tobacco industry and share what they find with others. The sessions also cover the health effects of tobacco, passive smoking, nicotine addiction and the economic cost of smoking. Sessions one and two include video clips followed by individual and group-based quizzes, and discussion activities where students learn about the harmful and addictive nature of smoking and methods used by tobacco companies to encourage young people to smoke. Students are provided with a workbook to record their answers. In session three, they then use this information to 'spread the word' in a group presentation to their class, in a medium of their choice such as through drama or song.
**b) Family component**
A ten-page A5 booklet accompanying the Year 7 lessons, designed to stimulate discussions about smoking between parents and students at home. The booklet contained the following interactive activities: 1) a repeat of the quiz questions students completed themselves in class to enable them to test their parents' knowledge about areas such as the health effects of smoking; 2) consideration of new information about the marketing practices employed by the tobacco industry, with particular focus on how young people might be targeted; 3) an opportunity for

For peer review only

students to give advice to other young people in various scenarios they might find themselves in relating to smoking.
**c) Year 8 booster component**<br>A one hour interactive session for 'off the shelf' delivery in Year 8 focusing on tobacco marketing strategies from the perspective of a tobacco industry executive and marketing company, as well as a health campaigner, both seen through the eyes of a teenager and reported direct to camera in the form of a social media blog. Students are asked a series of questions relating to the tobacco industry at key moments in the storyline. Two further optional activities are: 1) writing a slogan for a billboard poster, advertising a fake cigarette brand, in order to raise their awareness of how tobacco companies may portray smoking to young people; 2) writing a 'tweet' about the tobacco industry enabling students to reflect on their learning and consider their personal thoughts and feelings about this.

For peer review only

Supplementary file 2: Themes and sub-themes identified in qualitative data from Year 7 and Year 8 students and teachers delivering to both groups

**Theme**	**Sub-themes**
Teachers' preparedness to deliver *Operation Smoke Storm**	• Previous experience teaching tobacco control<br>• Preparation before delivering the intervention<br>• Confidence to deliver the intervention<br>• Use of the teaching resources provided
Raised awareness	• New information learnt<br>- Prior knowledge; What's in a cigarette; Health effects; Tobacco industry; Understanding the message; Adding to knowledge learnt in Year 7 (booster)<br>• Impact of new information<br>- Realising the seriousness of smoking; Perceptions of smoking; Decision to Smoke; Using new information; Concerns and worries about others' smoking
Engagement with *Operation Smoke Storm*	• Views on the classroom-based sessions<br>- Messaging and storyline; Structure and timings; Formatting; Nature and variety of activities; Student ability<br>- Views on the booster session: Storyline and angle; Formatting; Following on from Year 7; Student ability; Nature and variety of activities<br>• Views on the family booklet<br>- How it was used; Discussions; Students' raised awareness of tobacco-related issues; Parental attitudes and raised awareness of tobacco-related issues; Timing
Extending *Operation Smoke Storm*	• E-cigarettes<br>- Students' knowledge; Discussions had in class; Students' perceptions and usage of e-cigs<br>• Peer pressure

*Data for this theme solely from teachers. Remaining themes represent data from students in Years 7 and 8 as well as teachers.

For peer review only

Table 1 Consolidated criteria for reporting qualitative studies (COREQ): 32-item checklist No Item Guide questions/description

Domain/item	Guide/ questions description	Author comments
Domain 1: Research team and reflexivity Personal Characteristics		
1.Interviewer/facilitator	Which author/s conducted the interview or focus group?	Page 10 cites the paper (ref 9) with these details to save space in this manuscript.
2.Credentials	What were the researcher's credentials?	Focus groups were conducted by MB (PhD), AT (MSc), and LS (PhD). Interviews with teachers and parents were completed by AT and JT (PhD). All had been involved in the data collection conducted during the first stage and MB, AT and JT have previous experience of conducting qualitative interviews/focus groups.
3.Occupation	What was their occupation at the time of the study?	MB – Assistant Professor, LS – Associate Professor, JT – Research Fellow on the trial, and AT – Research Assistant on the trial
4.Gender	Was the researcher male or female?	Both males and females were involved in the data collection.
5.Experience and training	What experience or training did the researcher have?	All researchers had been involved in the qualitative data collected during the first stage (ref 9 cited in the paper on page 10). MB, JT and AT are all experienced in leading interviews and focus groups, which they have conducted previously. The more experienced researchers led the data collection, where LS acted as a facilitator due to having less experience in collecting qualitative data.
6.Relationship established	Was a relationship established prior to study commencement?	Relationship was established with staff at the schools prior to the study commencing. LS, MB, JT and AT delivered a training/introductory session

For peer review only

		to teachers that would be involved in intervention delivery which outlined the resource and the research components (data collection). Information sheets outlined the purpose of the study including the qualitative evaluation (referred to on page 10, where ref 9 is cited). The researchers introduced themselves and the purpose of the interviews, focus groups prior to commencing them. They gave individuals the chance to ask questions prior to them completing the consent forms and before starting data collection
7.Participant knowledge of the interviewer	What did the participants know about the researcher? e.g. personal goals, reasons for doing the research	Prior to beginning the qualitative evaluation (i.e. focus groups, interviews) the researchers reiterated the purpose of the research/interview/focus groups and allowed individuals to ask questions before beginning. They were informed that we were carrying out the study to find out what they thought of the resource and how it could be improved. Outlined in ref 9 cited on page 10.
8.Interviewer characteristics	What characteristics were reported about the interviewer/facilitator? e.g. Bias, assumptions, reasons and interests in the research topic	None, other than that we were interested in hearing what their views on the resource, booster and family components, particularly to hear whether/how it could be improved. This was outlined prior to conducting any of the qualitative interviews. We encouraged participants to share their honest views and that there were not any right/wrong answers.
Domain 2: study design<br>Theoretical framework		
9.Methodological orientation	What methodological	Framework approach (pages

and Theory	orientation was stated to underpin the study? e.g. grounded theory, discourse analysis, ethnography, phenomenology, content analysis	11-12). We used an inductive approach to identify themes.
10.Participant selection: Sampling	How were participants selected? e.g. purposive, convenience, consecutive, snowball	Pages 10-11 (again, we refer to reference 9 for readers to find more details as this was similar to earlier work).
11.Method of approach	How were participants approached? e.g. face-to-face, telephone, mail, email	Pages 10-11 outlines the approach and reference 9 is cited for readers to refer to for further details. Teachers invited students to express an interest in taking part in a focus group. Teachers were approached via email and parents were invited to return a slip if they wanted to take part in paired parent-student interview. This was provided with a letter that detailed the study.
12.Sample size	How many participants were in the study?	Pages 10-11 detail the final numbers of participants that took part: 16 Year 7 students , 51 year 8 students, 16 teachers and 9 parent-student interviews were conducted.
13.Non-participation	How many people refused to participate or dropped out? Reasons?	N/A those who volunteered and subsequently turned up/completed the focus group or interview.
14.Setting of data collection	Where was the data collected? e.g. home, clinic, workplace	Pages 10-11 (procedures for focus groups and interviews same as those in earlier work and thus reference 9 cited to save space).Focus groups conducted in schools, interviews with teachers via phone or at the school and parent-student ones conducted at individual's home.
15.Presence of non-participants	Was anyone else present besides the participants and researchers?	No.
16.Description of sample	What are the important characteristics of the sample? e.g. demographic data, date	Pages 10-11 report numbers according to gender and school (i.e. school 1/2)

For peer review only

17.Interview guide	Were questions, prompts, guides provided by the authors? Was it pilot tested?	Pages 10-11 report topics covered by the guides
18.Repeat interviews	Were repeat interviews carried out? If yes, how many?	N/A
19.Audio/visual recording	Did the research use audio or visual recording to collect the data?	Reported on pages 10-11 – all data audio-recorded only.
20.Field notes	Were field notes made during and/or after the interview or focus group?	N/A as audio-recorded
21.Duration	What was the duration of the interviews or focus group?	Covered on pages 10-11.
22.Data saturation	Was data saturation discussed?	Data analysis section on pages 12 reports saturation
23.Transcripts returned	Were transcripts returned to participants for comment and/or correction	N/A – accuracy of transcriptions checked against audio-recordings.
Domain 3: analysis and findings		
24.Number of data coders	How many data coders coded the data?	The data analysis section on pages 11-12 reports who was involved in the analysis and derivation of the themes.
25.Description of the coding tree	Did authors provide a description of the coding tree?	The data analysis section on pages 11-12 covers the themes identified as does Appendix 1.
26.Derivation of themes	Were themes identified in advance or derived from the data?	The analysis process on pages 11-12 outlines how themes were derived inductively.
27.Software	What software, if applicable, was used to manage the data?	NVivo 11 software was used and this is reported on page 12.
28.Participant checking	Did participants provide feedback on the findings?	No.
29.Quotations presented	Were participant quotations presented to illustrate the themes / findings? Was each quotation identified? e.g. participant number	Quotes are presented in the results section to illustrate the findings.
30.Data and findings consistent	Was there consistency between the data presented and the findings?	Yes
31.Clarity of major themes	Were major themes clearly presented in the findings?	Yes in the Results and Discussion sections.
32.Clarity of minor themes	Is there a description of diverse cases or discussion of minor themes?	Diverse cases are reflected in the results and considered in the Discussion section.

For peer review only

STROBE Statement—checklist of items that should be included in reports of observational studies

	Item No	Recommendation
**Title and abstract**	1	(*a*) Indicate the study's design with a commonly used term in the title or the abstract (page 1)
		(*b*) Provide in the abstract an informative and balanced summary of what was done and what was found (pages 2-3)
**Introduction**		
Background/rationale	2	Explain the scientific background and rationale for the investigation being reported (pages 5-6)
Objectives	3	State specific objectives, including any prespecified hypotheses (page 6)
**Methods**		
Study design	4	Present key elements of study design early in the paper (pages 6 (end of introduction, elaboration in methods section, pages 6-7)
Setting	5	Describe the setting, locations, and relevant dates, including periods of recruitment, exposure, follow-up, and data collection (pages 6-7)
Participants	6	(*a*) *Cohort study*—Give the eligibility criteria, and the sources and methods of selection of participants. Describe methods of follow-up<br>*Case-control study*—Give the eligibility criteria, and the sources and methods of case ascertainment and control selection. Give the rationale for the choice of cases and controls (pages 6-8)<br>*Cross-sectional study*—Give the eligibility criteria, and the sources and methods of selection of participants
		(*b*) *Cohort study*—For matched studies, give matching criteria and number of exposed and unexposed<br>*Case-control study*—For matched studies, give matching criteria and the number of controls per case
Variables	7	Clearly define all outcomes, exposures, predictors, potential confounders, and effect modifiers. Give diagnostic criteria, if applicable (pages 6-9)
Data sources/ measurement	8*	For each variable of interest, give sources of data and details of methods of assessment (measurement). Describe comparability of assessment methods if there is more than one group (pages 6-9)
Bias	9	Describe any efforts to address potential sources of bias (N/A)
Study size	10	Explain how the study size was arrived at (page 9)
Quantitative variables	11	Explain how quantitative variables were handled in the analyses. If applicable, describe which groupings were chosen and why (pages 8-10)
Statistical methods	12	(*a*) Describe all statistical methods, including those used to control for confounding (pages 8-10)
		(*b*) Describe any methods used to examine subgroups and interactions
		(*c*) Explain how missing data were addressed (Table 1)
		(*d*) *Cohort study*—If applicable, explain how loss to follow-up was addressed<br>*Case-control study*—If applicable, explain how matching of cases and controls was addressed (pages 8-9)<br>*Cross-sectional study*—If applicable, describe analytical methods taking account of

For peer review only

	sampling strategy
	(*e*) Describe any sensitivity analyses (pages 9-10: Bayes)

Continued on next page

For peer review only

For peer review only - http://bmjopen.bmj.com/site/about/guidelines.xhtml

**Results**		
Participants	13*	(a) Report numbers of individuals at each stage of study—eg numbers potentially eligible, examined for eligibility, confirmed eligible, included in the study, completing follow-up, and analysed (pages 8-10, 15 [including Table 2])
		(b) Give reasons for non-participation at each stage (pages 8-10)
		(c) Consider use of a flow diagram
Descriptive data	14*	(a) Give characteristics of study participants (eg demographic, clinical, social) and information on exposures and potential confounders (pages 15-16 [including Table 2])
		(b) Indicate number of participants with missing data for each variable of interest (pages 15-16 – Table 2)
		(c) *Cohort study*—Summarise follow-up time (eg, average and total amount)
Outcome data	15*	*Cohort study*—Report numbers of outcome events or summary measures over time
		Case-control study—Report numbers in each exposure category, or summary measures of exposure (reported throughout the results sections i.e. intervention schools versus control schools)
		Cross-sectional study—Report numbers of outcome events or summary measures
Main results	16	(*a*) Give unadjusted estimates and, if applicable, confounder-adjusted estimates and their precision (eg, 95% confidence interval). Make clear which confounders were adjusted for and why they were included (reported throughout the results section)
		(*b*) Report category boundaries when continuous variables were categorized (results on pages 19-20 clearly describe results according to the category boundaries measured)
		(*c*) If relevant, consider translating estimates of relative risk into absolute risk for a meaningful time period
Other analyses	17	Report other analyses done—eg analyses of subgroups and interactions, and sensitivity analyses (Bayes factors results reported on page 17)
**Discussion**		
Key results	18	Summarise key results with reference to study objectives (Opening paragraph of discussion, pages 21-22)
Limitations	19	Discuss limitations of the study, taking into account sources of potential bias or imprecision. Discuss both direction and magnitude of any potential bias (pages 23-24 in particular highlight limitations of the study)
Interpretation	20	Give a cautious overall interpretation of results considering objectives, limitations, multiplicity of analyses, results from similar studies, and other relevant evidence (page 24 – final paragraph)
Generalisability	21	Discuss the generalisability (external validity) of the study results (pages 23-24 discuss generalisability)
**Other information**		
Funding	22	Give the source of funding and the role of the funders for the present study and, if applicable, for the original study on which the present article is based (page 25 funding stated)

*Give information separately for cases and controls in case-control studies and, if applicable, for exposed and unexposed groups in cohort and cross-sectional studies.

Note: An Explanation and Elaboration article discusses each checklist item and gives methodological background and published examples of transparent reporting. The STROBE checklist is best used in conjunction with this article (freely available on the Web sites of PLoS Medicine at http://www.plosmedicine.org/, Annals of Internal Medicine at

For peer review only

http://www.annals.org/, and Epidemiology at http://www.epidem.com/). Information on the STROBE Initiative is available at www.strobe-statement.org.

For peer review only

For peer review only - http://bmjopen.bmj.com/site/about/guidelines.xhtml

BMJ Open

Evaluation of a novel intervention providing insight into the tobacco industry to prevent the uptake of smoking in school-aged children: a mixed-methods study

Journal:	*BMJ Open*
Manuscript ID	bmjopen-2017-018031.R1
Article Type:	Research
Date Submitted by the Author:	03-Aug-2017
Complete List of Authors:	Szatkowski, Lisa; University of Nottingham<br>Taylor, John; University of Nottingham<br>Fuller, Amy; University of Nottingham<br>Lewis, Sarah; University of Nottingham<br>Wu, Qi; University of York, Department of Health Sciences<br>Parrott, Steve; University of York<br>McNeill, Ann; King’s College London, UK Centre for Tobacco Control Studies, National Addiction Centre, Institute of Psychiatry<br>Britton, John; University of Nottingham/ UK Centre for Tobacco and Alcohol Studies, Division of Epidemiology<br>Bauld, Linda; University of Stirling, Institute for Social Marketing<br>Jones, Laura; University of Birmingham, Public Health, Epidemiology & Biostatistics<br>Bains, Manpreet; University of Nottingham, Epidemiology and Public Health
**Primary Subject Heading**:	Addiction
Secondary Subject Heading:	Smoking and tobacco
Keywords:	PUBLIC HEALTH, Smoking, Prevention, Young people

SCHOLARONE™
Manuscripts

For peer review only

Evaluation of a novel intervention providing insight into the tobacco industry to prevent the uptake of smoking in school-aged children: a mixed-methods study

Lisa Szatkowski[1], John Taylor[1], Amy Fuller[1], Sarah Lewis[1], Qi Wu[2], Steve Parrott[2], Ann McNeill[3], John Britton[1], Linda Bauld[4], Laura L Jones[5], Manpreet Bains[1]

[1,2,3,4,5]UK Centre for Tobacco and Alcohol Studies;

[1]University of Nottingham, Division of Epidemiology and Public Health, Clinical Sciences Building, Nottingham City Hospital, Nottingham, UK, NG5 1PB;

[2]Department of Health Sciences, University of York, Seebohm Rowntree Building, Heslington, York, UK, YO10 5DD;

[3]King's College London, National Addictions Centre, Institute of Psychiatry, Psychology and Neuroscience, Addiction Sciences Building, 4 Windsor Walk, Denmark Hill, London, UK, SE5 8BB;

[4]Institute for Social Marketing, 3Y1, University of Stirling, Stirling, UK, FK9 4LA;

[5]Public Health, Epidemiology and Biostatistics, School of Health and Population Sciences, College of Medical and Dental Sciences, University of Birmingham, Edgbaston, Birmingham, UK, B15 2TT.

Correspondence to: Dr Manpreet Bains: manpreet.bains@nottingham.ac.uk

For peer review only

ABSTRACT

Objectives: Evidence from the US Truth® campaign suggests that interventions focusing on tobacco industry practices and ethics may be effective in preventing youth smoking uptake. We developed, piloted and evaluated a school-based intervention based on this premise.

Methods: Exploratory study Students in Years 7–8 (aged 11–13) in two UK schools received *Operation Smoke Storm*, comprising three 50-minute classroom-based sessions in Year 7, an accompanying family booklet and a 1-hour classroom-based booster session in Year 8. We compared the risk and odds of ever smoking and susceptibility to smoking in Year 8 students in study schools post-intervention compared with students in control schools. Focus groups and interviews with students, teachers and parents evaluated the acceptability of the intervention.

Results: In intervention schools the combined prevalence of ever smoking and susceptibility increased from 18.2% in Year 7 to 33.8% in Year 8. There was no significant difference in the odds of a□Year 8 student in an intervention school being an ever smoker or susceptible never smoker compared with controls [adjusted OR 1.28, 95%CI 0.83-1.97, p=0.263] and no significant difference in the odds of ever smoking (aOR 0.82, 95%CI 0.42-1.58, p=0.549). Teachers highlighted differences by academic ability in how well the messages presented were understood. Use of the family component was low but was received positively by parents who engaged with it.

For peer review only

For peer review only - http://bmjopen.bmj.com/site/about/guidelines.xhtml

Conclusions: *Operation Smoke Storm* is an acceptable resource for delivering smoking-prevention education but it does not appear to have reduced smoking and susceptibility.

For peer review only

For peer review only - http://bmjopen.bmj.com/site/about/guidelines.xhtml

Strengths and limitations

- We used a mixed-methods design that enabled triangulation of quantitative and qualitative data to strengthen the internal and external validity of the findings.
- Conclusions are based on data from only two intervention schools, which served relatively more affluent and ethnically white populations than the national average.
- The comparison with external, non-randomised control data meant there were significant differences between the characteristics of students in intervention and control schools.
- Logistical difficulties meant we were unable to link students' responses at baseline and follow-up, though smoking behaviours differed little between intervention and control schools at baseline and analyses were adjusted for confounders measured at follow-up.

For peer review only - http://bmjopen.bmj.com/site/about/guidelines.xhtml

INTRODUCTION

In the UK, nearly 40% of adult smokers start to smoke regularly before the age of 16[1] and those who start at an early age are more likely to die from a smoking-attributable cause[2]. Therefore, preventing young people from smoking is an important public health priority and school-based approaches provide an opportunity to reach large numbers of young people. Existing school-based approaches to smoking prevention differ in theoretical approach, design and mode of delivery. However, there is no evidence that any one approach is more superior to another, and little conclusive evidence that school-based prevention interventions have anything beyond short-term effects[3–5]. In the only UK study to show significant benefit, training school pupils to initiate conversations about smoking with their peers has been shown to reduce smoking uptake up to two years later[6], though since the publication of this study approaching a decade ago there have been substantial changes in public attitudes towards smoking as well as in the tobacco control and education environments.

In the United States the mass media *Truth*® campaign has demonstrated some success in encouraging young people not to smoke, focusing on the ethics and exploitative tactics of the tobacco industry[7–9]. Its acceptability and effectiveness has been recognised as worth exploring further in school settings[5]. Previously we have reported results of a preliminary qualitative evaluation amongst Year 7 students (aged 11-12) in two UK schools of the acceptability of a novel school-based intervention, *Operation Smoke Storm* (OSS), based on the premise of *Truth*®[10]. Initially, *OSS* comprised three 50-minute multimedia interactive teaching sessions, developed by *Kick It*, who deliver the National Health Service (NHS) Stop Smoking Service for

For peer review only - http://bmjopen.bmj.com/site/about/guidelines.xhtml

several London boroughs[11]. Further description of this intervention is given in supplementary file 1.

In focus groups conducted after the delivery of *OSS* students reported enjoying the intervention and acquiring new knowledge about smoking and the tobacco industry, which seemed to strengthen their aversion to smoking[10]. In one-to-one interviews teachers expressed confidence delivering the 'off the shelf' resource, although they highlighted a need for the package to be flexible and not dependent on lesson length, teacher confidence or expertise[10]. Following this feedback, Year 7 lessons were refined by the research team alongside *Kick It*, primarily to correct technical issues and to increase flexibility and provide teachers with more guidance to help them facilitate discussions regardless of their own level of knowledge. The intervention was also extended to include a family booklet to complement the Year 7 lessons to encourage parents to talk to their children about smoking and a 'booster' session for use with Year 8 students (aged 12-13) to reinforce the anti-smoking message. These family and booster components are described in supplementary file 1. Here we report quantitative and qualitative data evaluating the acceptability and effectiveness of the full intervention package.

METHODS

Quantitative evaluation

Collection of baseline and follow-up data

For peer review only - http://bmjopen.bmj.com/site/about/guidelines.xhtml

Six secondary schools in the UK East Midlands region were approached and two agreed to participate in delivering and evaluating OSS. The characteristics of the two schools where OSS was delivered are described in detail elsewhere[10]. Personal, Social, Health and Economic Education (PSHE) teachers delivered the first intervention component to all Year 7 students in both schools (n=585) in autumn 2013. Before and after intervention delivery all students were asked to complete an anonymous questionnaire to gather information on their socio-demographic characteristics as well as smoking behaviours and attitudes. Students were asked if they had ever smoked, as well as a set of three previously-validated questions to assess their susceptibility to smoking[12]:

1) do you think that you will try a cigarette soon? (yes/no)
2) if one of your best friends were to offer you a cigarette, would you smoke it? (definitely yes/ probably yes/ probably not/ definitely not)
3) do you think you will smoke a cigarette at any time during the next year? (definitely yes/ probably yes/ probably not/ definitely not)

Students were classified as non-susceptible if they answered 'no' to the first question 'and 'definitely not' to questions two and three. Students giving other combinations of responses were classified as susceptible.

One year later, in autumn 2014, the booster session was delivered to the same students, then in Year 8 (n=538). In School 1 PSHE specialists delivered the booster; 40-minute lessons meant they needed two sessions to cover the material. In School 2, changes in the organisation of PSHE meant that the booster was instead delivered by science teachers; lessons here were one hour in length and the material was delivered

For peer review only - http://bmjopen.bmj.com/site/about/guidelines.xhtml

in a single session. An anonymous questionnaire was administered after the booster session to gather data on smoking behaviours and attitudes and socio-demographic characteristics.

In autumn 2014 the refined Year 7 intervention component was also delivered to the new cohort of Year 7 students (n=350) in School 1 only, and these students were given the new family booklet to take home. Changes in the delivery of PSHE in School 2 meant that they were not able to accommodate delivery of the Year 7 sessions. Questionnaire data were collected at the end of the sessions to gain information about the acceptability of the revised intervention and family component.

Collection of control data from a non-randomised comparison group

Given some difficulty in recruiting schools, and in order to minimise costs, we chose to use external control data collected as part of another study just prior to ours. The Nottingham School Smoking Survey collected data from students in eight schools local to the study area in Spring 2011, 2012 and 2013 (though not all schools participated in every wave). The primary aim of this survey was to evaluate changes in young people's smoking behaviour following the introduction of point-of-sale tobacco display legislation[13,14]. By mid-2013 data were available on current smoking and susceptibility to smoking in Year 7 and Year 8 for two successive cohorts of students (i.e. students who were in Year 7 in 2011 and Year 8 in 2012 and students who were in Year 7 in 2012 and Year 8 in 2013).

Statistical analysis

For peer review only - http://bmjopen.bmj.com/site/about/guidelines.xhtml

All data management and analysis was carried out using Stata v13 (StataCorp, College Station, TX). Logistic regression was used to compare the self-reported odds of a combined outcome of ever smoking and susceptibility to smoking, plus ever smoking on its own, in Year 8 students after the delivery of the booster session to the odds amongst Year 8 students in the two combined cohorts of students in control schools, using a multilevel model to adjust for clustering with the effect of school modelled as a random intercept. Due to difficulties in linking students' responses to the Year 7 and Year 8 questionnaires in intervention schools, odds ratios could not be adjusted for differences between intervention and control groups at baseline. However, models were adjusted for socio-demographic variables using data collected in Year 8 and smoking behaviour at Year 7 was compared between intervention and control schools to quantify any differences. Unfortunately, a comparable measure of deprivation was not available across intervention and control schools. Therefore, a proxy indicator of deprivation was created, considering students in the most deprived quintile of the Index of Multiple Deprivation (IMD) in the control schools and those who reported being eligible for free school meals in the intervention schools as deprived relative to all others. Given the exploratory nature of the study, we have not applied a correction for multiple hypothesis testing but, instead, have presented results with 95% confidence intervals (95% CIs) and p-values in order to allow the reader to evaluate the findings fully. We also calculated unadjusted and adjusted risk differences (using the 'adjrr' post-estimation command in Stata) to compare intervention and control schools.

For peer review only - http://bmjopen.bmj.com/site/about/guidelines.xhtml

For peer review only

The non-randomised study was not intended to be fully powered but was instead planned as an exploratory study of the potential effectiveness of the intervention. A pre-study power calculation, based on estimates of the likely achieved sample size in intervention and control schools and the self-reported prevalence of ever smoking and susceptibility amongst Year 8 students, suggested that we would be able to estimate the risk difference to within 6.6% i.e. if the observed effect was 6.7% or greater the confidence intervals would preclude the possibility of no effect or a negative effect of the intervention. This effect size was consistent with the size of effect that a subsequent cluster-randomised controlled trial would be powered to detect, and in line with the size of effect used to power the ASSIST study[6]. For each of our outcomes (ever smoking and susceptibility to smoking, plus ever smoking on its own) we also calculated Bayes factors under three different scenarios in order to assess whether our data provided substantial evidence for or against the null hypothesis: 1) assuming a maximum odds ratio of 2 i.e. a doubling of never smokers in intervention compared to control schools, taking hypothesised values uniformly distributed between 0 and the maximum as plausible values; 2) assuming a plausible predicted odds ratio of 2 and taking hypothesised values in a normal distribution around this value; 3) assuming a plausible predicted odds ratio of 2 and taking hypothesised values in a half normal distribution around this value. A Bayes factor of 3 or more was taken as substantial evidence against the null hypothesis and 1/3 or less as evidence for the null.

We have followed the STROBE statement in reporting the results of this study.

Qualitative evaluation

For peer review only - http://bmjopen.bmj.com/site/about/guidelines.xhtml

Focus group and interview procedures

The qualitative evaluation comprised focus groups with Year 7 and Year 8 students, interviews with teachers who delivered the Year 7 sessions and the Year 8 booster session, and paired Year 7 student-parent interviews to evaluate the family booklet, each guided by a semi-structured interview schedule. We used the same procedures as described previously[10]. In summary, we conducted two gender-specific focus groups with Year 7 students in the one school (School 1) that delivered the revised sessions (16 students in total – 8 male, 8 female) and eight focus groups with Year 8 students across the two schools (51 students in total – 25 male, 26 female). Students shared their views on the sessions and their awareness of and attitudes towards the tobacco industry and smoking. Both Year 7 focus groups lasted for 26 minutes and Year 8 focus groups lasted for 24 minutes on average (range 11–35 minutes). All Year 7 and Year 8 teachers who delivered part of the intervention were invited by email to be interviewed about its acceptability and effectiveness; ten Year 7 teachers and six Year 8 teachers took part (four from School 1, two from School 2, interviews lasted 26 minutes on average [range 19-33 minutes]). The family booklet was accompanied by a letter inviting parents to express an interest in participating in a paired student-parent interview to explore their views. These interviews sought students' and parents' views on the booklet and how they engaged with it. An inconvenience allowance (£15 high-street voucher) was offered to each pair who participated (n = 9). Interviews took place in participants' home or on school premises according to individual preference (lasted 23 minutes on average and ranged between 13 – 33 minutes).

For peer review only - http://bmjopen.bmj.com/site/about/guidelines.xhtml

Data analysis

Analysis procedures were similar to those used previously[10], which followed the framework approach[15,16]. Digital audio-recordings were transcribed verbatim. A sample of focus group and interview transcripts was read initially (by AT and JT) to identify initial codes, themes and sub-themes and any within- or between-group differences (school and gender). As in our earlier work, codes identified from the focus groups, teacher interviews and student-parent interviews were similar (apart from teachers' interview data identifying a theme about preparation to deliver the intervention) and thus all Year 7 data were analysed together and similarly all Year 8 data. Initial themes and sub-themes were discussed between the researchers (AT, JT, MB, LS) to reach consensus on an initial analytical framework. This framework was applied and refined following analysis of the remaining transcripts and until the point of data saturation. Data were then indexed according to the final framework and charted into matrices according to each theme to facilitate synthesis and interpretation.

Similar themes were identified for both the Year 7 and Year 8 intervention components, and these supported those reported in our initial evaluation[10]: *Teachers' preparedness to deliver OSS*; *Raised awareness*; *Engagement with the intervention*; and *Options for extending the resource* (see supplementary file 2 for details of themes). Qualitative findings with respect to the Year 7 sessions were similar to those reported previously[10] and the amendments made to correct technical issues, increase flexibility and provide teachers with more guidance were positively received.

For peer review only - http://bmjopen.bmj.com/site/about/guidelines.xhtml

Therefore, the qualitative findings presented here focus on evaluation of the family booklet and Year 8 booster session.

Ethics and consent

The study received ethical approval from the University of Nottingham Medical School Research Ethics Committee (reference 13122012 CHS EPH Smoking). Parents of students in both Year 7 and Year 8 were sent a letter informing them about *OSS* and the accompanying academic evaluation, approximately three weeks prior to delivery. They were asked to return an opt-out slip if they did not want their child to complete a questionnaire or to participate in a focus group. Students were able to opt out of questionnaire completion and were under no obligation to volunteer for focus groups. Written informed consent was obtained from participants prior to data collection.

For peer review only

RESULTS

Did *OSS* have an impact on smoking behaviour?

Completed questionnaires were received from 445 Year 8 students in intervention schools and 1,692 Year 8 students in control schools; Table 1 describes students' characteristics.

Table 1: Characteristics of Year 8 students in intervention and control schools

	Intervention schools, n (%)	Control schools, n (%)	p-value for difference[a]
**Total number of completed questionnaires received**	445	1692	
**Sex**			
Male	200 (44.9)	843 (49.8)	0.482
Female	216 (48.5)	843 (49.8)	
Missing	29 (6.5)	6 (0.4)	
**Ethnic group**			
White	368 (82.7)	1309 (77.4)	<0.001
Non-white	27 (6.1)	220 (13.0)	
Missing	50 (11.2)	163 (9.6)	
**Parental smoking**			
Neither	302 (67.9)	1123 (66.4)	0.031
At least one	106 (23.8)	516 (30.5)	
Missing	37 (8.3)	53 (3.1)	
**Sibling smoking**			
None	365 (82.0)	1461 (86.4)	0.852
At least one	43 (9.7)	178 (10.5)	
Missing	37 (8.3)	53 (3.1)	
**Smoking in the home**			
Not allowed	369 (82.9)	1460 (80.4)	<0.001
Allowed	36 (7.6)	375 (16.3)	
Missing	42 (9.4)	57 (3.4)	
**Number of friends who smoke**			
None	289 (64.9)	734 (43.4)	<0.001
One or two	48 (10.8)	236 (14.0)	
Three or more	18 (4.0)	254 (15.0)	
Missing	90 (20.2)	468 (27.7)	
**Rebelliousness and sensation seeking**[17]			
Low	225 (50.6)	870 (51.4)	0.661
High	176 (39.6)	715 (42.3)	
Missing	44 (9.9)	107 (6.3)	
**Academic performance (self-perceived)**			
Excellent or good	313 (70.3)	1228 (72.6)	0.372
Average or below average	92 (20.7)	406 (24.0)	
Missing	40 (9.0)	58 (3.4)	
**Eligible for free school meals**			
No	374 (84.0)	Not collected	N/A
Yes	25 (5.6)		

For peer review only - http://bmjopen.bmj.com/site/about/guidelines.xhtml

Missing	46 (10.3)		
**Index of Multiple Deprivation (IMD) quintile**			
Least deprived	Not collected	375 (22.2)	N/A
2		160 (9.5)	
3		282 (16.7)	
4		240 (14.2)	
Most deprived		261 (15.4)	
Missing		374 (22.1)	

[a] excluding missing data

As expected, given the non-randomised nature of the study there were significant differences between students in intervention and control schools. In control schools a greater proportion of students were of non-white ethnicity, had parents who smoked, reported smoking was allowed in their home, had more friends who smoked and were ever smokers themselves.

Table 2 shows the odds of a student being a susceptible never smoker and/or an ever smoker in Year 7 and Year 8 in the two intervention schools compared to control schools. After adjusting for significant confounders, there were no differences in ever smoking and susceptibility to smoking between intervention and control schools in Year 7. In Year 8, after adjusting for significant confounders, the odds of a student in an intervention school being an ever smoker or susceptible never smoker were 28% higher than the odds for a student in a control school, though this difference was not statistically significant (adjusted OR 1.28, 95% CI 0.83-1.97, p=0.263). The adjusted risk difference suggested a non-significant 4.1% higher prevalence of ever smoking and susceptibility to smoking in intervention schools. Students in intervention schools were slightly less likely to have ever smoked compared to students in control schools, though again the difference was not statistically significant (adjusted OR 0.82, 95% CI 0.42-1.58, p=0.549). The adjusted risk difference suggested a non-significant 2.0% lower prevalence of ever smoking in intervention schools.

For peer review only - http://bmjopen.bmj.com/site/about/guidelines.xhtml

Table 2: Odds ratios and adjusted risk differences for smoking outcomes in intervention compared to control schools

	Prevalence (%)		Unadjusted			Adjusted*		
	Students in intervention schools	Students in control schools	Odds of outcome in intervention vs. control schools OR (95% CI)	p-value	Risk difference % (95% CI)	Odds of outcome in intervention vs. control schools OR (95% CI)	p-value	Risk difference % (95% CI)
**Year 7 (before intervention delivery)**								
**Ever smoker or susceptible never smoker**	18.2 (92/505)	22.9 (351/1530)	0.82 (0.43-1.55)	0.536	-4.7 (-15.3-5.9)	1.74 (0.54-5.56)	0.351	5.9 (-13.8-2.6)
**Ever smoker**	2.4 (12/505)	6.4 (98/1530)	0.38 (0.13-1.08)	0.070	-4.0 (-6.9-1.2)	1.22 (0.13-11.3)	0.858	0.4 (-9.9-10.8)
**Year 8 (after intervention delivery)**								
**Ever smoker or susceptible never smoker**	33.8 (145/429)	30.9 (504/1631)	1.17 (0.70-1.95)	0.556	2.9 (-4.0-9.8)	1.28 (0.83-1.97)	0.263	4.1 (-0.5-8.6)
**Ever smoker**	7.9 (34/429)	10.7 (175/1631)	0.80 (0.32-1.98)	0.622	-2.8 (-7.8-2.1)	0.82 (0.42-1.58)	0.549	-2.0 (-5.4-1.4)

*Adjusted for: perceived academic ability; rebelliousness; sibling smoking; parental smoking; and whether smoking is allowed in the family home.

For peer review only - http://bmjopen.bmj.com/site/about/guidelines.xhtml

Bayes factors for the combined outcome were 1/3 or lower under each of the three scenarios tested, suggesting that our data provide substantial evidence for the null hypothesis of no positive effect of the intervention. Bayes factors for ever smoking were all close to one, suggesting that our data are insensitive and unable to distinguish between the alternative and null hypotheses.

What did students, teachers and parents think about *OSS*?

Students broadly liked *OSS*; 77.1% of Year 7 students said that the revised Year 7 sessions were very good or okay and 72.4% of Year 8 students evaluated the booster session similarly. Qualitative data from Year 8 focus groups showed the booster session was well received and that most students bought into the storyline (Table 3a,b).

Of the 61.6% of Year 7 students who reported receiving the family booklet and taking it home, 43.0% said they showed it to their mother or another adult female, 21.5% reported showing to their father or another adult male and 24.4% said that they did not show the booklet to anyone. Very few reported having completed activities with a parent or carer. Even though Year 7 students and parents who were interviewed endorsed the family booklet as a way to improve knowledge and initiate conversations around smoking (Table 3c,d), our qualitative data also indicated that often the booklet was not used as intended – many students simply did not show the booklet to their parents or realise the booklet was for them to complete with their parents (Table 3e,f).

For peer review only - http://bmjopen.bmj.com/site/about/guidelines.xhtml

Table 3: What did students, teachers and parents think about *OSS*?

a) *It was really good. It was something different and I liked it.* (School 2, F)

b) *They're [the videos] really cool because I like when that girl went on a mission. That was, kind of like, interesting because I was like, "What is she going to do next?* (School 1, F)

c) *I learned something, I didn't know about all the additives if you like; and the sneaky way that the big companies and the amount of money involved and all of that really.* (School 1, Parent 1)

d) *We've discussed it since and had a chat about it. We were talking about it the other day, weren't we, things like the booklet and things like that and talking about what we now know about it. It was building on really what you'd done in [Drug Abuse Resistance Education] DARE at primary, wasn't it, just taking it a bit further*. (School 1, Parent 7)

e) *My tutor didn't really explain what it actually was about, so I didn't know I actually had to do anything with it, that's why I didn't show my mum.* (School 1, Parent 3)

f) *That's why I just thought, "oh, it's for my parents, it's not for me."* (School 1, F)

Did *OSS* change students' knowledge and attitudes about smoking?

69.3% of Year 7 students and 45.0% of Year 8 students thought that *OSS* had made it less likely that they would ever try a cigarette. Students displayed some changes in knowledge and attitudes over the course of the study (Table 4).

For peer review only - http://bmjopen.bmj.com/site/about/guidelines.xhtml

Table 4: Mean Likert scale responses (1=strongly agree, 5=strongly disagree)

	How far do you agree with the following statements? (mean+SD for statements 1-3; median+IQR for statement 4)			p-value*
	Baseline	After Year 7 lessons in Phase 1	After Year 8 lessons in Phase 2	
1) Companies that make cigarettes only try to attract customers aged 18+	2.30 (1.04)	2.85 (1.22)	3.47 (1.07)	<0.001
2) Companies that make cigarettes sell dangerous products, but still operate in a fair and decent way	2.79 (0.95)	2.80 (1.04)	2.95 (0.95)	0.030
3) Smoking is not that serious compared with other drugs young people use	3.06 (1.13)	3.20 (1.16)	3.24 (1.09)	0.034
4) Nicotine in cigarettes is one of the most addictive drugs that people use	2 (1-3)	2 (1-2)	2 (1-3)	<0.001

*ANOVA F test for normally distributed variables, Kruskal-Wallis test for non-normally distributed variables

Qualitative findings from Year 8 students and teachers suggest the booster session raised awareness of the harmful effects of tobacco and some students showed an appreciation of why and how the tobacco industry might target young people (Table 5a,b). However, teachers mentioned that not all students understood this message and highlighted differences in the extent to which students of higher and lower academic abilities could remember the new information and complete the activities (Table 5c,d).

Table 5: Did *OSS* change students' knowledge and attitudes about smoking?

a) *I didn't know about like all the effects until this year, and it's just like, it just shows you what actually smoking does. It just opened my eyes a bit.* (School 1, F)

b) *If they target to young people and try and get to young people, then they will get more money, 'cause there'll be more people getting addicted to it.* (School 2, F)

c) *Do you know that little clip where the boss is being very subtle going, 'oh do you use social media?'. And, 'oh we could do brand placement. Oh but we're not allowed to.' And it was all very subtle…Yeah and do you know lower-ability pupils wouldn't have got that. I think that would have confused them, where the other pupils it wouldn't have.* (School 1, Teacher 1)

d) *So whenever they talked about like their Tweets for social media, they kind of went for, 'You shouldn't smoke, it's bad for you. You shouldn't smoke. Cigarettes have*

For peer review only - http://bmjopen.bmj.com/site/about/guidelines.xhtml

got all this stuff in them,' so kind of the obvious stuff from it, but they then don't take it that step further to think, like, should they be publicising it, yeah, taking that conversation a bit deeper. (School 1, Teacher 2)

DISCUSSION

This project was the first to formally evaluate a school-based smoking prevention intervention highlighting the ethics and exploitative tactics of the tobacco industry. The intervention was feasible to deliver in the classroom, was generally acceptable to teachers, students and parents and helped to raise awareness about smoking-related issues and the tobacco industry. However, there was no significant difference in the odds or risk of self-reported ever smoking and susceptibility to smoking in students who received *OSS* compared to students from local schools where the intervention was not delivered.

Synthesis of the quantitative and qualitative data offers potential suggestions as to why the intervention does not appear to be effective in preventing smoking uptake. In both the focus groups with Year 7 students reported previously[10], and those following delivery of the revised Year 7 sessions, students' interest and recall centred mainly on the chemical constituents of cigarettes and/or the health effects of smoking. There was some suggestion from teachers that concepts relating to tobacco marketing, particularly where they were mentioned more subtly, were too advanced for students of lower academic ability to fully grasp. Given that educational attainment is inversely associated with adolescent smoking[17], it might be that *OSS* failed to reach those students most likely to become smokers.

For peer review only - http://bmjopen.bmj.com/site/about/guidelines.xhtml

The prevalence of smoking amongst young people increases with age[18] and it might be that any effect of *OSS* on uptake is delayed beyond the follow-up period studied here. Many students reported that participation in *OSS* had made it less likely that they would try a cigarette, and there was evidence of increasing disagreement over time with statements such as ‘smoking is not that serious compared with other drugs that young people use’. These data are encouraging and, although these shifts in attitudes are not reflected in self-reported smoking and susceptibility in Year 8, the possibility remains that the impact of the intervention may become evident among these students in years to come.

The Year 8 students on whom the primary analysis is based received the original version of the Year 7 lessons that were subsequently revised. Therefore it is possible that the effect of the revised resources on smoking and/or susceptibility might have been different. However, given the fact that the majority of the changes made were to correct technical issues rather than changes to content, this is unlikely. In addition, Year 8 students had not received the family component of the intervention. However, few Year 7 students in the second phase of the study used the booklet as intended so it is unlikely that this would have a substantial effect on the outcomes.

The 95% CIs around the odds ratios quantifying differences in smoking behaviours between students in intervention and control schools were wide, and the adjusted risk differences were small. The direction of the point estimate for the odds of ever smoking tentatively suggests that exposure to *OSS* might reduce the odds of this outcome, although the odds ratio for the combined outcome of ever smoking plus susceptibility suggests an increase in odds. A reduction in ever smoking following

For peer review only - http://bmjopen.bmj.com/site/about/guidelines.xhtml

exposure to *OSS* would be encouraging, and with a larger sample size the precision of the effect estimates would improve and smaller effect sizes may be detected as statistically significant.

The study findings are based on data from only two schools and may not be generalisable to schools more widely, particularly with regard to students' ethnicity and deprivation. The non-randomised comparison meant there were significant differences between the characteristics of students in intervention and control schools, which we were not able to adjust for. Our conclusions also rely on self-reported data, even though measures such as ensuring students' anonymity were in place to encourage honest responses.

The use of topic guides and the rigorous analytical process of the framework approach counterbalanced any potential for biased interpretation in favour of the intervention. However, some Year 8 focus groups had a small number of participants meant there was a less than ideal group dynamic. Finally, the students, teachers and parents who took part in the focus groups and interviews were a self-selecting sample, which introduced potential for bias.

Despite there being no evidence of effectiveness in this study, there is scope for further work to understand whether the concept behind *OSS* is worth pursuing further. *OSS* as it stands is probably not suitable for use with students older than the Year 7 and Year 8 groups, but the concept might be effective if used as the basis of an age-appropriate intervention with older students who might be better able to engage with subtle messages about industry influences. Alternatively, *OSS* might usefully be

For peer review only - http://bmjopen.bmj.com/site/about/guidelines.xhtml

adapted to include fully differentiated activities and resources for use with different academic abilities. Given the erosion of PSHE within the curriculum, there is scope to understand whether *OSS* could be delivered effectively in other settings such as youth groups. Finally, further work is warranted to explore how to effectively engage parents and guardians more in supporting their child to remain smoke free.

DECLARATIONS

Acknowledgements: We thank the PSHE teaching leads, teachers and students at the schools who took part in this study, as well as the chair and members of the Study Steering Committee and Data Monitoring and Evaluation Committee for their valuable advice and support. We also thank Tomasz Letniowski and Toby Fairs-Billam at Kick It who developed *OSS* but who were independent of this evaluation.

Authors' contributions: LS, MB, SL, AM, JB, LLJ, LB, SP and QW designed the study. JT and AT led the focus groups and conducted the teacher interviews; MB, LS and LLJ observed the focus groups. JT and AT analysed the data with MB providing external validation of themes. LS and JT wrote the first draft of this manuscript. All authors made critical comments on subsequent drafts of the paper and have approved the final version.

Data sharing: Requests for access to data should be addressed to the corresponding author.

For peer review only - http://bmjopen.bmj.com/site/about/guidelines.xhtml

Funding: This project was funded by the National Institute for Health Research Public Health Research (NIHR PHR) Programme [grant number 11/3010/02]. The views and opinions expressed herein are those of the authors and do not necessarily reflect those of the NIHR PHR Programme or the Department of Health. The authors are members of the UK Centre for Tobacco and Alcohol Studies (UKCTAS), a UK Clinical Research Collaboration (UKCRC)-funded Public Health Research Centre of Excellence. Funding from the British Heart Foundation, Cancer Research UK, the Economic and Social Research Council, the Medical Research Council and the National Institute of Health Research, under the auspices of the UK Clinical Research Collaboration, is gratefully acknowledged.

Competing interests: Laura Jones receives personal fees from the National Centre for Smoking Cessation and Training, outside the submitted work. There are no other competing interests to report.

REFERENCES

1. Dawe F. *General Lifestyle Survey Overview. A Report of the 2011 General Lifestyle Survey*. Newport: Office for National Statistics; 2013. http://www.ons.gov.uk/ons/dcp171776_302558.pdf.

2. Department of Health. *Smoking Kills: A White Paper on Tobacco*. London: Department of Health; 1998. http://www.archive.official-documents.co.uk/document/cm41/4177/4177.htm.

3. Thomas R, McLellan J, Perera R. School-based programmes for preventing smoking. *Cochrane Database Syst Rev*. 2013;4(CD001293). doi:10.1002/14651858.CD001293.pub3.

4. Wiehe S, Garrison M, Christakis D, Ebel B, Rivara F. A systematic review of schoolbased smoking prevention trials with long-term follow-up. *J Adolesc Health*. 2005;36:162-169. doi:10.1016/j.jadohealth.2004.12.003.

For peer review only

5. National Institute for Health and Clinical Excellence. *School-Based Interventions to Prevent the Uptake of Smoking among Children and Young People (NICE Public Health Guidance 23)*. London: National Institute for Health and Clinical Excellence; 2010. http://www.nice.org.uk/guidance/PH23.

6. Campbell R, Starkey F, Holliday J, et al. An informal school-based peer-led intervention for smoking prevention in adolescence (ASSIST): a cluster randomised trial. *The Lancet*. 2008;371(9624):1595-1602. doi:10.1016/S0140-6736(08)60692-3.

7. The truth® campaign. http://www.protectthetruth.org/truthcampaign.htm. Accessed September 12, 2016.

8. Farrelly M, Healton C, Davis K, Messeri P, Hersey J, Haviland M. Getting to the Truth: Evaluating National Tobacco Countermarketing Campaigns. *Am J Public Health*. 2002;92:901-907.

9. Farrelly M, Davis K, Haviland M, Messeri P, Healton C. Evidence of a dose-response relationship between "truth" antismoking ads and youth smoking prevalence. *Am J Public Health Bull Edinb*. 2005;95:425-431. doi:10.2105/AJPH.2004.049692.

10. Taylor J, Taylor A, Lewis S, et al. A qualitative evaluation of a novel intervention using insight into tobacco industry tactics to prevent the uptake of smoking in school-aged children. *BMC Public Health*. 2016;16:539. doi:10.1186/s12889-016-3205-8.

11. Kick It. https://www.kick-it.org.uk/. Accessed September 12, 2016.

12. Pierce J, Choi W, Gilpin E, Farkas A, Merritt R. Validation of susceptibility as a predictor of which adolescents take up smoking in the United States. *Health Psychol*. 1996;15(5):355-361. doi:10.1037/0278-6133.15.5.355.

13. Spanopoulos D, Britton J, McNeill A, Ratschen E, Szatkowski L. Tobacco display and brand communication at the point of sale: implications for adolescent smoking behaviour. *Tob Control*. 2014;23(1):64-69. doi:10.1136/tobaccocontrol-2012-050765.

14. Bogdanovica I, Szatkowski L, McNeill A, Spanopoulos D, Britton J. Exposure to point-of-sale displays and changes in susceptibility to smoking: findings from a cohort study of school students. *Addict Abingdon Engl*. 2015;110(4):693-702. doi:10.1111/add.12826.

15. Ritchie J, Lewis J, Elam G. *Qualitative Research Practice: A Guide for Social Science Students and Researchers.* London: Sage; 2003.

16. Gale NK, Heath G, Cameron E, Rashid S, Redwood S. Using the framework method for the analysis of qualitative data in multi-disciplinary health research. *BMC Med Res Methodol*. 2013;13:117. doi:10.1186/1471-2288-13-117.

For peer review only - http://bmjopen.bmj.com/site/about/guidelines.xhtml

17. Amos A, Angus K, Bostock Y, Fidler J, Hastings G. *A Review of Young People and Smoking in England*. Public Health Research Consortium; 2009. http://phrc.lshtm.ac.uk/papers/PHRC_A7-08_Final_Report.pdf.

18. Fuller E (ed). *Smoking, Drinking and Drug Use among Young People in England in 2014*. Leeds: Health and Social Care Information Centre; 2015. http://www.hscic.gov.uk/catalogue/PUB17879/smok-drin-drug-youn-peop-eng-2014-rep.pdf.

For peer review only

For peer review only - http://bmjopen.bmj.com/site/about/guidelines.xhtml

Supplementary file 1: Outline of the Operation Smoke Storm intervention

**a) Year 7 component**<br>*Operation Smoke Storm* is a web-based educational package designed for delivery by teachers as part of a school's Personal, Social, Health and Economic Education (PSHE) curriculum. Teachers are provided with detailed lesson plans for three 50-minute classroom sessions (although the material can also be delivered as one longer session). Multimedia presentations, streamed 'live' over the Internet from *Kick It's* servers, are used to guide teachers and students through the lessons. Students act as secret agents to uncover the tactics of the tobacco industry and share what they find with others. The sessions also cover the health effects of tobacco, passive smoking, nicotine addiction and the economic cost of smoking. Sessions one and two include video clips followed by individual and group-based quizzes, and discussion activities where students learn about the harmful and addictive nature of smoking and methods used by tobacco companies to encourage young people to smoke. Students are provided with a workbook to record their answers. In session three, they then use this information to 'spread the word' in a group presentation to their class, in a medium of their choice such as through drama or song.
**b) Family component**<br>A ten-page A5 booklet accompanying the Year 7 lessons, designed to stimulate discussions about smoking between parents and students at home. The booklet contained the following interactive activities: 1) a repeat of the quiz questions students completed themselves in class to enable them to test their parents' knowledge about areas such as the health effects of smoking; 2) consideration of new information about the marketing practices employed by the tobacco industry, with particular focus on how

For peer review only

young people might be targeted; 3) an opportunity for students to give advice to other young people in various scenarios they might find themselves in relating to smoking.
**c) Year 8 booster component**<br>A one hour interactive session for 'off the shelf' delivery in Year 8 focusing on tobacco marketing strategies from the perspective of a tobacco industry executive and marketing company, as well as a health campaigner, both seen through the eyes of a teenager and reported direct to camera in the form of a social media blog. Students are asked a series of questions relating to the tobacco industry at key moments in the storyline. Two further optional activities are: 1) writing a slogan for a billboard poster, advertising a fake cigarette brand, in order to raise their awareness of how tobacco companies may portray smoking to young people; 2) writing a 'tweet' about the tobacco industry enabling students to reflect on their learning and consider their personal thoughts and feelings about this.

For peer review only

Supplementary file 2: Themes and sub-themes identified in qualitative data from Year 7 and Year 8 students and teachers delivering to both groups

Theme	Sub-themes
Teachers' preparedness to deliver *Operation Smoke Storm**	• Previous experience teaching tobacco control<br>• Preparation before delivering the intervention<br>• Confidence to deliver the intervention<br>• Use of the teaching resources provided
Raised awareness	• New information learnt<br>- Prior knowledge; What's in a cigarette; Health effects; Tobacco industry; Understanding the message; Adding to knowledge learnt in Year 7 (booster)<br>• Impact of new information<br>- Realising the seriousness of smoking; Perceptions of smoking; Decision to Smoke; Using new information; Concerns and worries about others' smoking
Engagement with *Operation Smoke Storm*	• Views on the classroom-based sessions<br>- Messaging and storyline; Structure and timings; Formatting; Nature and variety of activities; Student ability<br>- Views on the booster session: Storyline and angle; Formatting; Following on from Year 7; Student ability; Nature and variety of activities<br>• Views on the family booklet<br>- How it was used; Discussions; Students' raised awareness of tobacco-related issues; Parental attitudes and raised awareness of tobacco-related issues; Timing
Extending *Operation Smoke Storm*	• E-cigarettes<br>- Students' knowledge; Discussions had in class; Students' perceptions and usage of e-cigs<br>• Peer pressure

*Data for this theme solely from teachers. Remaining themes represent data from students in Years 7 and 8 as well as teachers.

For peer review only

Table 1 Consolidated criteria for reporting qualitative studies (COREQ): 32-item checklist No Item Guide questions/description

Domain/item	Guide/ questions description	Author comments
Domain 1: Research team and reflexivity Personal Characteristics		
1.Interviewer/facilitator	Which author/s conducted the interview or focus group?	Page 10 cites the paper (ref 9) with these details to save space in this manuscript.
2.Credentials	What were the researcher's credentials?	Focus groups were conducted by MB (PhD), AT (MSc), and LS (PhD). Interviews with teachers and parents were completed by AT and JT (PhD). All had been involved in the data collection conducted during the first stage and MB, AT and JT have previous experience of conducting qualitative interviews/focus groups.
3.Occupation	What was their occupation at the time of the study?	MB – Assistant Professor, LS – Associate Professor, JT – Research Fellow on the trial, and AT – Research Assistant on the trial
4.Gender	Was the researcher male or female?	Both males and females were involved in the data collection.
5.Experience and training	What experience or training did the researcher have?	All researchers had been involved in the qualitative data collected during the first stage (ref 9 cited in the paper on page 10). MB, JT and AT are all experienced in leading interviews and focus groups, which they have conducted previously. The more experienced researchers led the data collection, where LS acted as a facilitator due to having less experience in collecting qualitative data.
6.Relationship established	Was a relationship established prior to study commencement?	Relationship was established with staff at the schools prior to the study commencing. LS, MB, JT and AT delivered a training/introductory session

For peer review only

		to teachers that would be involved in intervention delivery which outlined the resource and the research components (data collection). Information sheets outlined the purpose of the study including the qualitative evaluation (referred to on page 10, where ref 9 is cited). The researchers introduced themselves and the purpose of the interviews, focus groups prior to commencing them. They gave individuals the chance to ask questions prior to them completing the consent forms and before starting data collection
7.Participant knowledge of the interviewer	What did the participants know about the researcher? e.g. personal goals, reasons for doing the research	Prior to beginning the qualitative evaluation (i.e. focus groups, interviews) the researchers reiterated the purpose of the research/interview/focus groups and allowed individuals to ask questions before beginning. They were informed that we were carrying out the study to find out what they thought of the resource and how it could be improved. Outlined in ref 9 cited on page 10.
8.Interviewer characteristics	What characteristics were reported about the interviewer/facilitator? e.g. Bias, assumptions, reasons and interests in the research topic	None, other than that we were interested in hearing what their views on the resource, booster and family components, particularly to hear whether/how it could be improved. This was outlined prior to conducting any of the qualitative interviews. We encouraged participants to share their honest views and that there were not any right/wrong answers.
Domain 2: study design<br>Theoretical framework		
9.Methodological orientation	What methodological	Framework approach (pages

and Theory	orientation was stated to underpin the study? e.g. grounded theory, discourse analysis, ethnography, phenomenology, content analysis	11-12). We used an inductive approach to identify themes.
10.Participant selection: Sampling	How were participants selected? e.g. purposive, convenience, consecutive, snowball	Pages 10-11 (again, we refer to reference 9 for readers to find more details as this was similar to earlier work).
11.Method of approach	How were participants approached? e.g. face-to-face, telephone, mail, email	Pages 10-11 outlines the approach and reference 9 is cited for readers to refer to for further details. Teachers invited students to express an interest in taking part in a focus group. Teachers were approached via email and parents were invited to return a slip if they wanted to take part in paired parent-student interview. This was provided with a letter that detailed the study.
12.Sample size	How many participants were in the study?	Pages 10-11 detail the final numbers of participants that took part: 16 Year 7 students , 51 year 8 students, 16 teachers and 9 parent-student interviews were conducted.
13.Non-participation	How many people refused to participate or dropped out? Reasons?	N/A those who volunteered and subsequently turned up/completed the focus group or interview.
14.Setting of data collection	Where was the data collected? e.g. home, clinic, workplace	Pages 10-11 (procedures for focus groups and interviews same as those in earlier work and thus reference 9 cited to save space).Focus groups conducted in schools, interviews with teachers via phone or at the school and parent-student ones conducted at individual's home.
15.Presence of non-participants	Was anyone else present besides the participants and researchers?	No.
16.Description of sample	What are the important characteristics of the sample? e.g. demographic data, date	Pages 10-11 report numbers according to gender and school (i.e. school 1/2)

For peer review only

17.Interview guide	Were questions, prompts, guides provided by the authors? Was it pilot tested?	Pages 10-11 report topics covered by the guides
18.Repeat interviews	Were repeat interviews carried out? If yes, how many?	N/A
19.Audio/visual recording	Did the research use audio or visual recording to collect the data?	Reported on pages 10-11 – all data audio-recorded only.
20.Field notes	Were field notes made during and/or after the interview or focus group?	N/A as audio-recorded
21.Duration	What was the duration of the interviews or focus group?	Covered on pages 10-11.
22.Data saturation	Was data saturation discussed?	Data analysis section on pages 12 reports saturation
23.Transcripts returned	Were transcripts returned to participants for comment and/or correction	N/A – accuracy of transcriptions checked against audio-recordings.
Domain 3: analysis and findings		
24.Number of data coders	How many data coders coded the data?	The data analysis section on pages 11-12 reports who was involved in the analysis and derivation of the themes.
25.Description of the coding tree	Did authors provide a description of the coding tree?	The data analysis section on pages 11-12 covers the themes identified as does Appendix 1.
26.Derivation of themes	Were themes identified in advance or derived from the data?	The analysis process on pages 11-12 outlines how themes were derived inductively.
27.Software	What software, if applicable, was used to manage the data?	NVivo 11 software was used and this is reported on page 12.
28.Participant checking	Did participants provide feedback on the findings?	No.
29.Quotations presented	Were participant quotations presented to illustrate the themes / findings? Was each quotation identified? e.g. participant number	Quotes are presented in the results section to illustrate the findings.
30.Data and findings consistent	Was there consistency between the data presented and the findings?	Yes
31.Clarity of major themes	Were major themes clearly presented in the findings?	Yes in the Results and Discussion sections.
32.Clarity of minor themes	Is there a description of diverse cases or discussion of minor themes?	Diverse cases are reflected in the results and considered in the Discussion section.

For peer review only

STROBE Statement—checklist of items that should be included in reports of observational studies

	Item No	Recommendation
**Title and abstract**	1	(*a*) Indicate the study's design with a commonly used term in the title or the abstract (page 1)
		(*b*) Provide in the abstract an informative and balanced summary of what was done and what was found (pages 2-3)
**Introduction**		
Background/rationale	2	Explain the scientific background and rationale for the investigation being reported (pages 5-6)
Objectives	3	State specific objectives, including any prespecified hypotheses (page 6)
**Methods**		
Study design	4	Present key elements of study design early in the paper (pages 6 (end of introduction, elaboration in methods section, pages 6-7)
Setting	5	Describe the setting, locations, and relevant dates, including periods of recruitment, exposure, follow-up, and data collection (pages 6-7)
Participants	6	(*a*) *Cohort study*—Give the eligibility criteria, and the sources and methods of selection of participants. Describe methods of follow-up<br>*Case-control study*—Give the eligibility criteria, and the sources and methods of case ascertainment and control selection. Give the rationale for the choice of cases and controls (pages 6-8)<br>*Cross-sectional study*—Give the eligibility criteria, and the sources and methods of selection of participants
		(*b*) *Cohort study*—For matched studies, give matching criteria and number of exposed and unexposed<br>*Case-control study*—For matched studies, give matching criteria and the number of controls per case
Variables	7	Clearly define all outcomes, exposures, predictors, potential confounders, and effect modifiers. Give diagnostic criteria, if applicable (pages 6-9)
Data sources/ measurement	8*	For each variable of interest, give sources of data and details of methods of assessment (measurement). Describe comparability of assessment methods if there is more than one group (pages 6-9)
Bias	9	Describe any efforts to address potential sources of bias (N/A)
Study size	10	Explain how the study size was arrived at (page 9)
Quantitative variables	11	Explain how quantitative variables were handled in the analyses. If applicable, describe which groupings were chosen and why (pages 8-10)
Statistical methods	12	(*a*) Describe all statistical methods, including those used to control for confounding (pages 8-10)
		(*b*) Describe any methods used to examine subgroups and interactions
		(*c*) Explain how missing data were addressed (Table 1)
		(*d*) *Cohort study*—If applicable, explain how loss to follow-up was addressed<br>*Case-control study*—If applicable, explain how matching of cases and controls was addressed (pages 8-9)<br>*Cross-sectional study*—If applicable, describe analytical methods taking account of

For peer review only

For peer review only - http://bmjopen.bmj.com/site/about/guidelines.xhtml

	sampling strategy
	(*e*) Describe any sensitivity analyses (pages 9-10: Bayes)

Continued on next page

For peer review only

For peer review only - http://bmjopen.bmj.com/site/about/guidelines.xhtml

**Results**		
Participants	13*	(a) Report numbers of individuals at each stage of study—eg numbers potentially eligible, examined for eligibility, confirmed eligible, included in the study, completing follow-up, and analysed (pages 8-10, 15 [including Table 2])
		(b) Give reasons for non-participation at each stage (pages 8-10)
		(c) Consider use of a flow diagram
Descriptive data	14*	(a) Give characteristics of study participants (eg demographic, clinical, social) and information on exposures and potential confounders (pages 15-16 [including Table 2])
		(b) Indicate number of participants with missing data for each variable of interest (pages 15-16 – Table 2)
		(c) *Cohort study*—Summarise follow-up time (eg, average and total amount)
Outcome data	15*	*Cohort study*—Report numbers of outcome events or summary measures over time
		Case-control study—Report numbers in each exposure category, or summary measures of exposure (reported throughout the results sections i.e. intervention schools versus control schools)
		Cross-sectional study—Report numbers of outcome events or summary measures
Main results	16	(*a*) Give unadjusted estimates and, if applicable, confounder-adjusted estimates and their precision (eg, 95% confidence interval). Make clear which confounders were adjusted for and why they were included (reported throughout the results section)
		(*b*) Report category boundaries when continuous variables were categorized (results on pages 19-20 clearly describe results according to the category boundaries measured)
		(*c*) If relevant, consider translating estimates of relative risk into absolute risk for a meaningful time period
Other analyses	17	Report other analyses done—eg analyses of subgroups and interactions, and sensitivity analyses (Bayes factors results reported on page 17)
**Discussion**		
Key results	18	Summarise key results with reference to study objectives (Opening paragraph of discussion, pages 21-22)
Limitations	19	Discuss limitations of the study, taking into account sources of potential bias or imprecision. Discuss both direction and magnitude of any potential bias (pages 23-24 in particular highlight limitations of the study)
Interpretation	20	Give a cautious overall interpretation of results considering objectives, limitations, multiplicity of analyses, results from similar studies, and other relevant evidence (page 24 – final paragraph)
Generalisability	21	Discuss the generalisability (external validity) of the study results (pages 23-24 discuss generalisability)
**Other information**		
Funding	22	Give the source of funding and the role of the funders for the present study and, if applicable, for the original study on which the present article is based (page 25 funding stated)

*Give information separately for cases and controls in case-control studies and, if applicable, for exposed and unexposed groups in cohort and cross-sectional studies.

Note: An Explanation and Elaboration article discusses each checklist item and gives methodological background and published examples of transparent reporting. The STROBE checklist is best used in conjunction with this article (freely available on the Web sites of PLoS Medicine at http://www.plosmedicine.org/, Annals of Internal Medicine at

For peer review only

http://www.annals.org/, and Epidemiology at http://www.epidem.com/). Information on the STROBE Initiative is available at www.strobe-statement.org.

For peer review only

For peer review only - http://bmjopen.bmj.com/site/about/guidelines.xhtml
